# Comparative genome mining and metabolomics reveal divergent NRPS-derived fengycin biosynthetic gene clusters in *Bacillus halotolerans*

**DOI:** 10.1093/femsmc/xtag037

**Published:** 2026-06-22

**Authors:** Mokhtar Salah Eddine Belhadj, Omar Messaoudi, Mohamed Yousfi, Amina Bakhrouf

**Affiliations:** Laboratory of Analysis, Treatment and Valorization of Pollutants of the Environmental and Products, Faculty of Pharmacy, University of Monastir, Monastir 68140, Tunisia; Laboratoire des Sciences Fondamentales (LSF), Université Amar Telidji de Laghouat, Route de Ghardaïa, BP 37G, Laghouat, Laghouat, 03000, Algeria; Research Unit of Medicinal Plant (RUMP) Attached to Center of Biotechnology (CRBt, 3000, Constantine), Laghouat, 03000, Algeria; Department of Biology, Faculty of Science, University of Amar Telidji, Laghouat, 03000, Algeria; Laboratory of Applied Microbiology in Food and Environment, Abou Bekr Belkaïd University, Tlemcen, 13000, Algeria; Laboratoire des Sciences Fondamentales (LSF), Université Amar Telidji de Laghouat, Route de Ghardaïa, BP 37G, Laghouat, Laghouat, 03000, Algeria; Laboratory of Analysis, Treatment and Valorization of Pollutants of the Environmental and Products, Faculty of Pharmacy, University of Monastir, Monastir 68140, Tunisia

**Keywords:** *Bacillus halotolerans*, comparative genomics, untargeted metabolomics, fengycin biosynthetic gene cluster, antimicrobial natural products

## Abstract

Microbial multidrug resistance is a major public health concern, underscoring the urgent need for new antimicrobial natural products. In this study, strain F11, identified as *Bacillus halotolerans*, was selected based on its strong antimicrobial activity and taxonomic identification. Whole-genome sequencing revealed a single circular chromosome of 4.15 Mb with a GC content of 43.82%, encoding 4122 predicted proteins. Pangenome analysis identified 17 unique genes. Genome mining predicted 10 biosynthetic gene clusters (BGCs), including a complete fengycin cluster. Comparative analyses using BiG-SCAPE/CORASON and clinker revealed evolutionary divergence within the fengycin BGCs, including those identified in *B. halotolerans* F11 and *B. halotolerans* HMB20199. This divergence was further supported by NRPS substrate specificity predictions, which revealed two amino acid variations at positions 6 and 8 in the predicted fengycin decapeptide of strain *B. halotolerans* F11 compared to the canonical sequence. In contrast, *B. halotolerans* HMB20199 exhibited a mosaic fengycin–iturin hybrid organization, characterized by an extended NRPS assembly line comprising 19 modules. Furthermore, untargeted metabolomic profiling of *B. halotolerans* F11 detected 9719 metabolites, of which 3453 were successfully annotated. Integration of genomic and metabolomic datasets enabled the correlation of two compounds—bacillaene and bacillibactin—with their corresponding BGCs. However, the lack of detection of fengycin, surfactin, and subtilosin A was attributable to methodological constraints. Collectively, these findings expand our understanding of *B. halotolerans* strains as promising genomic reservoirs of novel NRPS-derived lipopeptides and highlight Algerian Sahara soils as a valuable source of antimicrobial natural products.

## Teaser

Divergent NRPS-derived fengycin biosynthetic gene clusters were identified through an integrative genomic-metabolomic analysis of *Bacillus halotolerans* F11 and related strains.

## Introduction

The global rise of multidrug-resistant (MDR) microorganisms poses a serious public health challenge, particularly those grouped under the ESKAPE pathogens (*Enterococcus faecium, Staphylococcus aureus, Klebsiella pneumoniae, Acinetobacter baumannii, Pseudomonas aeruginosa*, and *Enterobacter* spp.), which not only exhibit resistance to multiple antibiotic classes but also withstand biocides and disinfectants through diverse mechanisms (De Oliveira et al. 2020, Belay et al. 2024). These MDR microorganisms spread across the human–animal–environment interface, affecting food and agricultural sectors and underscoring the need for integrated control strategies under the One Health framework (Pérez-Rodríguez and Mercanoglu 2019).

One promising avenue to address this challenge lies in the search for novel microbial natural products (NPs) from underexplored environments such as the Algerian Sahara, as these ecosystems harbor microorganisms with unique biosynthetic pathways capable of producing structurally diverse and potent metabolites (Messaoudi et al. 2022a). In particular, species belonging to the genus *Bacillus* are well known for producing an extensive arsenal of antimicrobial compounds, including cyclic lipopeptides (CLPs), which stand out due to their biodegradability, relatively low toxicity, and environmental compatibility (Medeot et al. 2023, Ranjan et al. 2023). Within this genus, *Bacillus halotolerans* has emerged as a promising candidate for the production of various CLPs, such as iturin, fengycin, bacillomycin, and surfactin (Shu et al. 2024). Among these CLPs, the decapeptide fengycin, characterized by a cyclic structure in which L-Tyr at position 3 and L-Ile at position 10 are linked through a lactone bond (Markelova et al. 2025). This lipopeptide has recently attracted considerable attention due to its broad range of biotechnological applications in agriculture, food preservation, and medicine (Hanif et al. 2019, Pérez-Rodríguez and Mercanoglu 2019, Wei et al. 2010).

Two complementary approaches are commonly used to study CLPs. The classical approach involves metabolite extraction and structural elucidation using techniques such as Liquid Chromatography coupled with High-resolution electrospray ionization mass spectrometry (LC-HRESIMS) and Nuclear Magnetic Resonance (NMR) ; however, this process is often labor-intensive and time-consuming (Messaoudi et al. 2022b). In contrast, genome mining enables the in silico identification of CLP biosynthetic gene clusters (BGCs), thereby accelerating the discovery of new metabolites (Kai et al. 2025). In this context, given the limited reports on the diversity of fengycin BGCs among *B. halotolerans* strains, the present study aimed to investigate divergent CLP BGCs that may encode previously uncharacterized fengycin. To achieve this objective, we performed: (i) prioritized screening to select *B. halotolerans* F11 from a collection of aerobic spore-forming bacteria based on antimicrobial activity and taxonomic identification; (ii) genome assembly and annotation of the *B. halotolerans* F11 strain; (iii) pangenome analysis to explore genomic diversity among *B. halotolerans* strains; (iv) comparative genomic analyses using BiG-SCAPE/CORASON and clinker to investigate potential divergence of detected CLP BGCs among *B. halotolerans* strains, including F11; (v) modular organization and substrate prediction of the NRPS megaenzyme encoded by potentially divergent CLP BGCs identified in *B. halotolerans* strains; and (vi) untargeted metabolomic profiling to associate detected metabolic features with predicted biosynthetic pathways in *B. halotolerans* F11.

## Material and methods

### Prioritized screening for the selection of aerobic spore-forming bacterial strains

During this study, spore-forming bacteria were isolated from four distinct environments in the Algerian Sahara region (Table S1) using a selective heat-shock method as described by Al-Humam (2016) (Supplementary Methods S1). All recovered isolates were subjected to primary antimicrobial screening against a panel of pathogenic microorganisms using the agar plug method. Briefly, isolates were grown on tryptic soy agar for 5 days at 30°C (Table S2), after which 6-mm agar plugs were transferred onto Mueller–Hinton agar for bacteria, and Sabouraud agar for yeasts, previously inoculated with target pathogens (Table S2). Plates were kept at 4°C for 4 h to allow diffusion of metabolites, then incubated at 37°C (bacteria), and 30°C (yeasts). Inhibition zones were measured after 24–48 h using a Haloes Caliper (IUL Instruments) (Bouzidi et al. 2023, Yahla et al. 2025).

The test panel included Gram-positive bacteria *S. aureus* (ATCC 25923), *Bacillus cereus* (ATCC 10876), and *Micrococcus luteus* (ATCC 15307), Gram-negative bacteria *P. aeruginosa* (ATCC 27853), *Escherichia coli* (ATCC 25922), and *K. pneumoniae* (ATCC 70603), *Salmonella typhi* (ATCC 14028), and *Yersinia enterocolitica* (ATCC 9610, as well as two yeasts: *Candida albicans* ATCC 10231 and *Candida albicans* ATCC 26790. Microbial suspensions were standardized to 0.5 McFarland following growth in brain–heart infusion broth for bacteria, and Sabouraud broth for yeasts.

Based on their inhibition profiles, five strains exhibiting the strongest antimicrobial activity were selected for further characterization.

### Molecular identification of selected isolates

Molecular identification of the selected strains was performed by 16S rRNA gene sequencing. Genomic DNA was extracted using the GF-1 Nucleic Acid Extraction Kit (Vivantis, Malaysia), and DNA quality and concentration were evaluated with a NanoDrop spectrophotometer (NanoDrop™ One, USA). Polymerase Chain Reaction (PCR) amplification was carried out using primers 27F (5′-AGAGTTTGATCCTGGCTCAG-3′) and 1492R (5′-CCGTCAATT CCTTTGAGTTT-3′) in a 50 μl reaction mixture containing 1.25 U Hot Start Taq DNA polymerase (Solis Biodyne, Estonia), 25–50 ng/μl template DNA, 0.3 μM of each primer, and 1.5 μM MgCl₂ (Dahmani et al. 2026). The PCR program included initial denaturation at 94°C for 12 min, followed by 30 cycles of 94°C for 30 s, 55°C for 30 s, and 72°C for 1 min 40 s, with a final extension at 72°C for 7 min. Amplification products were verified on 1.5% agarose gels stained with Midori Green Advance and visualized under UV light, with a 100-bp DNA ladder (Solis Biodyne, Estonia) as a size marker (Edwards et al. 1989). Purified PCR products (Vivantis Clean-Up Kit) were sequenced bidirectionally using the BigDye Terminator v3.1 kit on a 3730 XL Genetic Analyzer (Applied Biosystems). The resulting sequences were edited using BioEdit Alignment (v7) and compared with type strain sequences in the EZBioCloud/EZTaxon database for taxonomic identification (Thompson et al. 1994).

Based on antimicrobial profiles and taxonomic affiliation, among the five selected spores forming bacetria, one strains was selected for further evaluation as the most promising isolate.

### Whole-genome sequencing, assembly, comparative phylogenomics, and functional annotation

The genome of the selected strain was sequenced using the DNBSEQ platform at the Beijing Genomics Institute (Shenzhen, China). Genomic DNA was randomly fragmented by physico-chemical methods to construct three paired-end libraries of different sizes, in which both ends of each DNA fragment were sequenced to enhance assembly quality and read alignment. Low-quality reads were filtered out, and high-quality reads were de novo assembled using SOAPdenovo v1.05. A circular genome map was generated with Proksee for visualization.

For taxonomic placement, the assembled genome was analyzed using the Type (Strain) Genome Server (TYGS; https://tygs.dsmz.de) (Meier-Kolthoff et al. 2019). Intergenomic distances were computed, and a phylogenetic tree was reconstructed with FASTME v2.1.6.1 using subtree pruning and regrafting postprocessing. Branch support values were inferred from 100 pseudo-bootstrap replicates. Species-level relatedness was assessed through digital DNA–DNA hybridization (dDDH), calculated with the Genome-to-Genome Distance Calculator (http://ggdc.dsmz.de) using a generalized linear model. A dDDH threshold of 70% was applied for species delimitation. Average nucleotide identity (ANI) was computed with FastANI (Meier-Kolthoff et al. 2013).

Structural and functional annotation was carried out using Prokka on the Galaxy platform, enabling standardized gene prediction and functional assignment. For complementary analysis, the assembled genome was also submitted to the Rapid Annotation using Subsystems Technology (RAST; http://rast.nmpdr.org/) server, providing subsystem-based functional classification and insights into the organism’s metabolic potential (Brettin et al. 2015).

### Pangenome analysis

The pangenome analysis was performed between the selected strain complete genome and high-quality complete genomes of closely related strains belonging to the same species, retrieved from the RefSeq database in GenBank format. Each genome was annotated using Prokka on Galaxy to generate standardized GFF3 files.

Pan-genome analysis was then carried out using Roary, which clusters orthologous genes and produces a core gene alignment using MAFFT (Page et et al. 2015). This alignment was used to construct a maximum likelihood phylogenetic tree using FastTree2 (Price et al. 2010), implemented via the Galaxy Europe server (Jalili et al. 2020). The resulting phylogenetic tree was generated in nhx format (.nhx) and manually edited using Visual Studio Code to remove NHX tags. It was then saved in Newick format (.newick) for compatibility. The Newick tree was visualized using MEGA X and further integrated with metadata using Phandango. This enabled visualization of gene presence/absence patterns across the strains alongside phylogenetic relationships (Hadfield et al. 2018).

### BGC prediction and divergence analysis

The biosynthetic potential of the selected strain was analyzed using antiSMASH v8 with default parameters, enabling the identification and functional annotation of BGCs. The resulting GenBank (.gbk) files, together with those of the closest species, were used as input for BiG-SCAPE v1.1.2 in auto mode. Protein sequences predicted within each BGC were scanned for Pfam domains, and a similarity network was constructed using a Jaccard index cutoff of 0.3, clustering BGCs into gene cluster families (GCFs) (Navarro-Muñoz et al. 2020).

All predicted clusters were compared against the Minimum Information about a BGCs (MIBiG 4.0) repository (Zdouc et al. 2025; https://mibig.secondarymetabolites.org) to assess novelty. Similarity networks and comparison matrices were visualized using Cytoscape v3.8.2 (Shannon et al. 2003).

Furthermore, CORASON (CORe Analysis of Syntenic Orthologues to prioritize Natural product gene clusters), integrated within BiG-SCAPE, was used to investigate the evolutionary relationships among BGCs within selected GCFs, generating high-resolution multilocus phylogenies within and across families (Navarro-Muñoz et al. 2020). Finally, clinker and its companion visualization library clustermap.js were used to generate accurate and interactive gene cluster comparison figures (Gilchrist and Chooi 2021).

### Metabolomic characterization of selected strain

#### One strain-many compounds approach for secondary metabolite modulation

To enhance metabolite production in the selected strain, the one strain-many compounds (OSMAC) approach was applied. For this purpose, the strain was cultivated in different media, including nutrient agar, International Streptomyces Project 2 (ISP2), Methylophila medium (YIM38), and soybean medium (SM) (Table S2). The antimicrobial activity of each culture was then reassessed using the agar plug method (Smati et al. 2025). Subsequently, the medium yielding the highest antimicrobial activity was selected for downstream analyses, including minimum inhibitory concentration (MIC) determination. For this purpose, the strain was cultivated in 250 ml Erlenmeyer flasks containing 100 ml of the selected broth medium, inoculated with agar plugs from 48-h cultures. After 5 days of incubation, metabolites were extracted with ethyl acetate (1:1, v/v), centrifuged, and lyophilized to obtain crude extracts (Benbelkhir et al. 2022).

MIC values were determined by serial dilution in 96-well microplates against the pathogen panel, with MIC defined as the lowest concentration completely inhibiting visible growth (Ohikhena et al. 2017).

#### Untargeted metabolomic analysis of selected strain

The metabolomic analysis was performed for the selected strain by the BGI metabolomics platform, using a validated LC–MS/MS workflow under the same culture conditions that yielded potent antimicrobial activity, characterized by a low MIC value.

Serum samples were extracted with methanol:acetonitrile:water (4:2:1, v/v/v) containing internal standards, followed by vortexing, incubation at –20°C, and centrifugation at 25 000 × *g*. The supernatants were dried under vacuum, reconstituted in methanol:water (1:1, v/v), and centrifuged again to obtain the final extracts. A pooled quality control sample was prepared from aliquots of all extracts.

Metabolite separation was carried out on a Waters UPLC I-Class Plus system (Waters, USA) equipped with an ACQUITY BEH C18 column (1.7 µm, 2.1 × 100 mm, 45°C) and coupled to a Q Exactive Orbitrap mass spectrometer (Thermo Fisher Scientific, USA). Chromatographic separation employed gradient elution with either 0.1% formic acid in water (mobile phase A, positive mode) or 10 mM ammonium formate (mobile phase A, negative mode), and acetonitrile as mobile phase B, at a flow rate of 0.35 ml/min with an injection volume of 5 µl. MS data were acquired in full-scan mode (70–1000 m/z) at a resolution of 70 000, with MS/MS performed on the three most intense precursor ions.

Raw data were processed using Compound Discoverer 3.3 (Thermo Fisher Scientific) for peak extraction and annotation. Metabolite identification was achieved through comparison against the BGI Metabolome Database, mzCloud, and ChemSpider. The resulting data matrix, including peak intensities and metabolite annotations, was used for statistical and pathway enrichment analyses.

## Results and discussions

### Selection of *Bacillus halotolerans* F11 through a prioritized screening pipeline

To address the challenge of antimicrobial resistance, this study aimed to explore secondary metabolites produced by aerobic spore-forming bacteria isolated from Algerian Sahara soil as a promising source of antimicrobial agents. To achieve this goal, a single strain was selected from the collection using a prioritized pipeline strategy based primarily on two criteria: antimicrobial activity and taxonomic identification.

As the first step of this pipeline, twenty-six aerobic spore-forming bacterial strains were isolated from soil samples collected at different sites in the Algerian Sahara (Table S1), using a heat-shock treatment at 80°C, which selectively enriches spore-forming bacteria while eliminating heat-sensitive species (Methods S1 and Table S2) (Valenzuela et al. 2024). These strains were recovered from an unexplored desert ecosystem characterized by harsh environmental conditions (Messaoudi et al. 2020), which drive microbial metabolic pathways involved in antimicrobial compouds production with high biotechnological potential (Su et al. 2020, Pardo-Esté et al. 2024). Accordingly, all isolates were subjected to in vitro antimicrobial screening against a panel of Gram-positive and Gram-negative bacteria, as well as *C. albicans*. Based on their antimicrobial performance, five strains (F2, F8, F9, F11, and F12) were selected as the most promising candidates, and their antimicrobial activity results are summarized in Table 1.

**Table 1:** Antimicrobial activity (mm) of selected spore-forming bacteria against pathogenic microorganisms.

	F11	F8	F9	F12	F2
*P. aeruginosa*	11.5	11	10	11.5	15.5
*E. coli*	–	–	–	–	–
*Y. enterocolitica*	–	–	9	–	–
*B. cereus*	9.5	–	10	–	8.5
*S. aureus*	–	–	–	10	12
*M. luteus*	30	30	30	30	12
*K. pneumoniae*	–	–	–	–	–
*C. albicans 10*	10	–	–	-5	–
*C. albicans 26*	–	–	–	-5	–

– : No activity detected.

The results presented in Table 1 indicate that all five selected isolates exhibited antimicrobial activity against at least one of the tested microorganisms. Notably, all strains inhibited *P. aeruginosa*, a pathogen well known for its high resistance to many bioactive compounds (Fig. S1) (Messaoudi et al. 2020, Liu et al. 2022). However, no inhibition was observed against the other Gram-negative bacteria, *E. coli* and *K. pneumoniae*, except for a weak activity of isolate F9 against *Y. enterocolitica*. These findings are consistent with the well-known natural resistance of Gram-negative bacteria, largely attributed to their lipopolysaccharide-rich outer membrane, which acts as an effective protective barrier (Boudrahem et al. 2025, Messaoudi et al. 2020).

To further refine the selection for detailed genomic and metabolomic analysis, the five strains were then subjected to molecular identification using partial 16S rRNA gene sequencing. The obtained sequences were compared with closely related homologs using BLAST analysis, and the results are presented in Table 2.

**Table 2:** Molecular identification of the five selected strains based on partial 16S rRNA gene sequencing.

Strains	Sampling sites	Accession number	Closest types species	Similarity (%)
F2	Saline soil from Salt Mountain of Kaf El-Melh (Laghouat, Algeria)	PX471844	*B. licheniformis (ATCC14580^T^)*	99.30
			*B. haynesii (NRRL B-41327 ^T^)*	98.85
			*B. sonorensis (NBRC 101234 ^T^)*	98.85
F8	Sandy soil from Kef Mokrane (Laghouat, Algeria)	PX471845	*B. licheniformis (ATCC14580^T^)*	94.85
			*B. haynesii (NRRL B-41327 ^T^)*	94.70
			*B. sonorensis (NBRC 101234 ^T^)*	94.70
F11	Soil from Oued M’zi (Laghouat, Algeria)	PX471843	*B. halotolerans (DSM 8802 ^T^)*	98.55
			*B. rugosus (NRRL B-65559 ^T^)*	98.50
			*B. spizizenii (NBRC 101239 ^T^)*	98.50
F9	Industrial effluent water collected from a cooling tower of an Air Separation Unit	PX872935	*B. haynesii (NRRL B-41327 ^T^)*	97.75
			*B. licheniformis (DSM 13 ^T^)*	97.55
			*B. aerius (MTCC 7303^T^)*	97.00
F12	Soil from Oued M’zi (Laghouat, Algeria)	PX872936	*B. rugosus (NRRL B-65559 ^T^)*	97.75
			*B. cabrialesii(CCStamb A1 ^T^)*	97.55
			*B. subtilis (DSM 10 ^T^)*	97.40

Results presented on Table 2 indicated that strains F2 and F11 showed high sequence similarity to *B. licheniformis* (99.30%) and *B. halotolerans* (98.55%), respectively, while F8 and F9 were closely related to *B*. licheniformis (94.85%) and *B. haynesii* (97.75%). However, strain F12 showed the closest relationship to *B. rugosus*, with a sequence similarity of 97.75%.

The pronounced antimicrobial activity exhibited by strain F11 (Table 1), together with its close taxonomic affiliation with *B. halotolerans* (Table 2), a species well known for its significant biotechnological potential, led to its selection for further investigation. Accordingly, strain F11 was subjected to an integrated genomic and metabolomic analysis to characterize the biosynthetic features underlying its potent antimicrobial activity.

Detailed information on the morphological, physiological, and biochemical features of the selected strain is provided in Tables S3–S5.

### Whole-genome analysis, taxonomic affiliation, and functional annotation

**Figure 1:**
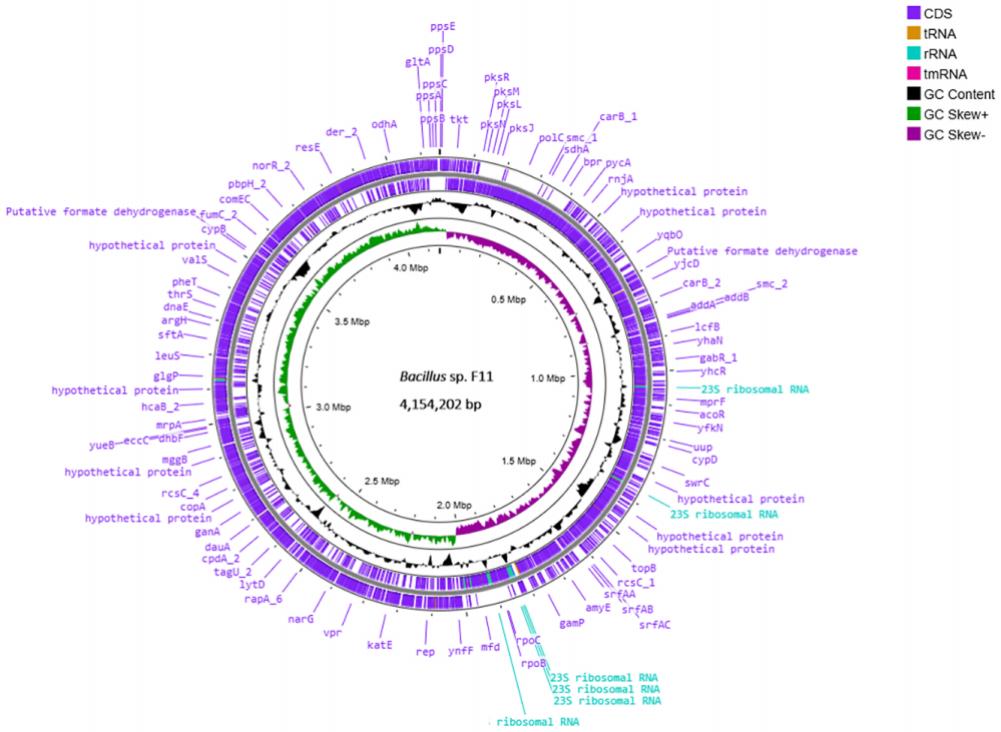
Circular annotated whole-genome map of *Bacillus* sp. F11

The complete genome of strain F11 was sequenced, assembled, and structurally annotated. The general genomic features of strain F11 are summarized in Table 3 and illustrated in Fig. 1.

**Table 3:** Structural annotation of the complete genome sequence of strain F11.

Characteristics	Statistical values
Contigs generated	1
Completeness	99.3%
Coverage	92.64%
Average sequencing depth(X)	76.11
Contamination	0.2%
Total length (bp)	4 154 202 bp
Number of coding DNA sequence (CDS)	4122
Number of rRNA	30
Number of tRNA	87
Number of tmRNA	1
N50	4 154 202
GC content (%)	43.82

The genome of strain F11 consists of a single circular chromosome comprising 4 154 202 base pairs, with a GC content of 43.82%. Structural annotation using the PROKKA pipeline identified a total of 4122 protein-coding sequences, in addition to 30 ribosomal RNA (rRNA) genes and 87 transfer RNA (tRNA) genes.

In order to resolve the taxonomic position of strain F11, given the limited resolution of 16S rRNA gene analysis at the species level, phylogenomic analyses and whole-genome-based comparisons were performed. For this purpose, a genome-based phylogenetic tree was constructed using the Genome BLAST Distance Phylogeny (GBDP) method implemented in the TYGS and inferred using the FastME algorithm (Meier-Kolthoff et al. 2019).

**Figure 2:**
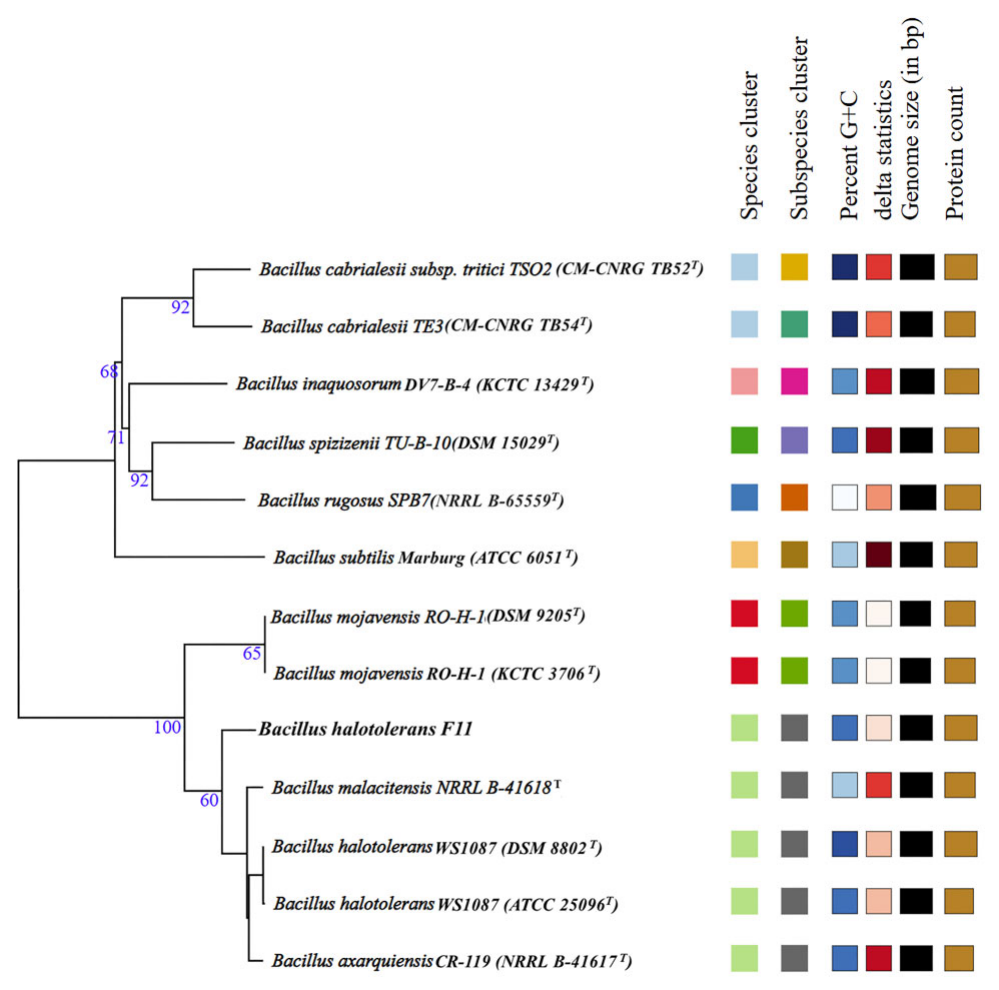
Phylogenomic tree of *Bacillus halotolerans* F11 and related type strains reconstructed using FastME 2.1.6.1 based on GBDP distances. Branch lengths are scaled using the d5 formula. Bootstrap support values (>60%) from 100 pseudo-replicates are shown above the branches, with an average support of 89.1%. The tree is midpoint-rooted.

The resulting tree (Fig. 2) placed strain F11 within a well-supported clade comprising *B. halotolerans* DSM 8802^T^, *B. halotolerans* ATCC 25096, “*B. malacitensis*” NRRL B-41618^T^, and “*B. axarquiensis*” NRRL B-41617^T^, with a bootstrap support value of 66%. These taxa represent members of the same species, as “*B. malacitensis*” and “*B. axarquiensis*” are later heterotypic synonyms of the validly published species *B. halotolerans* (Fig. 2) (Dunlap et al. 2016).

Furthermore, to confirm the species-level classification of strain F11, in silico genome-based similarity analyses were performed using dDDH and ANI. As shown in Table S6, strain F11 exhibited high dDDH values with *B. halotolerans* DSM 8802^T^ (89.1%) and *B. halotolerans* ATCC 25096 (89.1%). Comparable values were observed with “*B. malacitensis*” NRRL B-41618^T^ (90.5%) and “*B. axarquiensis*” NRRL B-41617^T^ (87.9%). All values exceeded the 70% threshold accepted for species delineation. In agreement, FastANI analyses (Table S7) yielded ANI values above the 95%–96% species boundary. Accordingly, based on the combined evidence from dDDH and ANI analyses (Tables S6 and S7), together with the phylogenomic results (Fig. 2), strain F11 was identified as *B. halotolerans*.

The complete genome sequence of *B. halotolerans* F11 strain has been deposited in the DDBJ/ENA/GenBank databases under the accession number JBREXF000000000. The version described in this paper is JBREXF020000000. The genome is associated with BioProject PRJNA1333569, BioSample SAMN51799607.

Furthermore, functional annotation of the *B. halotolerans* F11 genome was performed using the RAST server, which organized the predicted genes into functional subsystems. The obtained results are presented in Fig. S2.

RAST annotation results (Fig. S2) revealed that *B. halotolerans* F11 harbors a substantial number of genes related to sporulation (96 genes), stress response (19 genes), and defense (35 genes), in addition to eleven genes involved in aromatic compound degradation. Such gene sets likely contribute to its ecological adaptation and long-term survival in contaminated environments (Wu et al. 2021), which is consistent with its isolation origin from Oued M’zi (Laghouat, Algeria) (Table 2), a site impacted by industrial discharges.

### Pangenome analysis

The pangenome analysis was conducted to uncover genetic traits specific to *B. halotolerans* F11 and to clarify its phylogenetic relationship with closely related *B. halotolerans* strains based on core genome alignment. For this purpose, 24 complete *B. halotolerans* genomes were analyzed, and the cumulative pangenome statistics are presented in Fig. S3.

The pangenome analysis (Fig. S3), revealed a total of 8013 genes, of which 41% (3250 genes) constituted the core genome, representing conserved housekeeping functions essential for basic cellular processes (Matthews et al. 2024). The remaining 59% were distributed among soft core (2%), shell (16%), and cloud genes (41%), indicating substantial genomic plasticity. This large proportion of accessory genes supports an open pangenome structure, characteristic of environmentally adaptable bacterial species (Bach et al. 2022). The abundance of shell and cloud genes, often linked to horizontal gene transfer, stress tolerance, or niche-specific metabolic functions, highlights the adaptive potential of *B. halotolerans* species (Fiedoruk et al. 2021).

**Figure 3:**
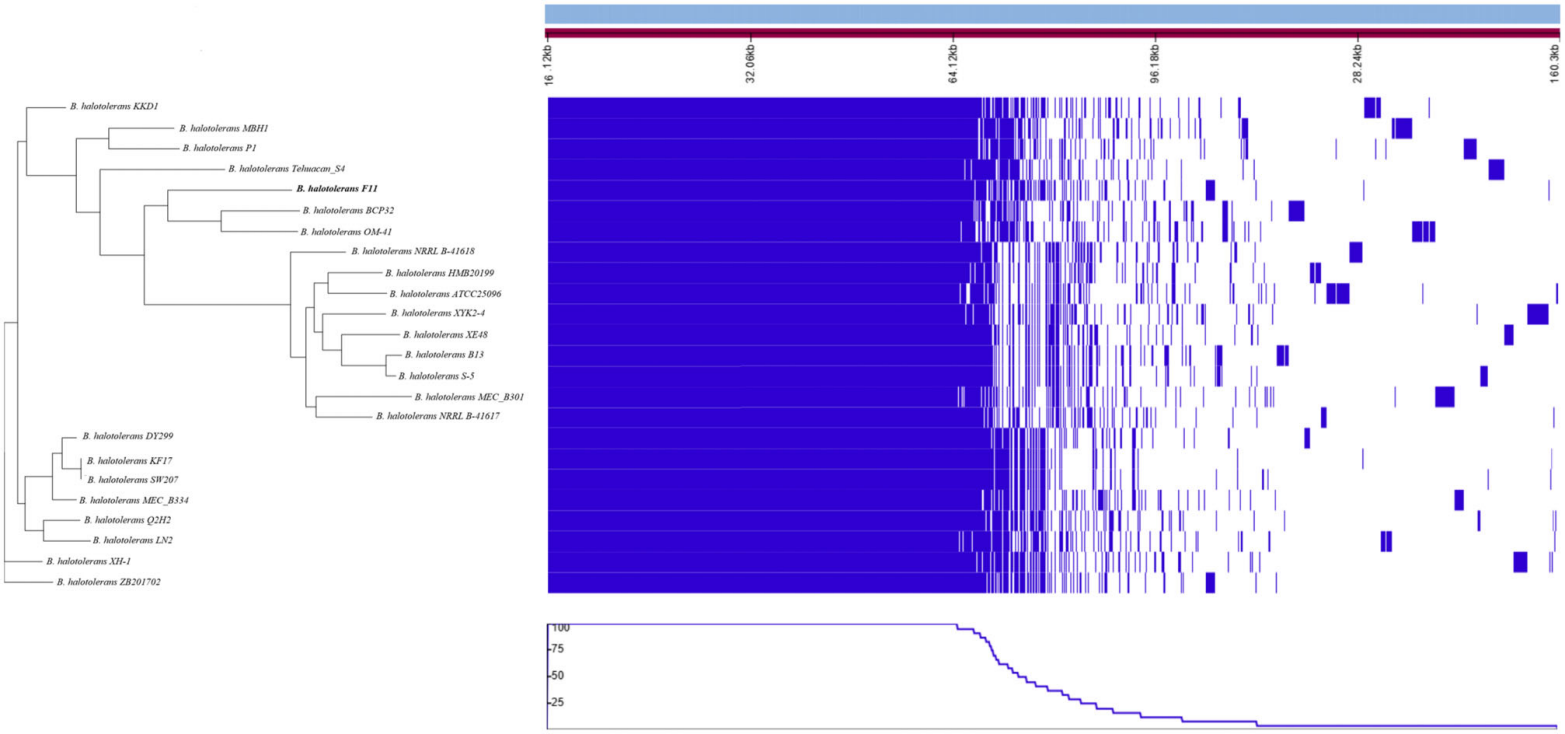
Phylogenomic tree based on core genome alignment of strain F11 and closely related *B. halotolerans* strains, with gene presence/absence profiles visualized using Phandango.

To further investigate the genetic diversity among *B. halotolerans* strains isolated from different geographic origins, a phylogenetic analysis based on the core genome alignment of 24 complete genomes was performed, with results presented in Fig. 3.

The phylogenetic tree clustered the 24 strains into two major clades (Fig. 3), which showed partial correlation with their geographic origins (Table S8). The first clade consists of eight strains, all isolated from rhizosphere soil samples collected in various regions of China, indicating high genomic similarity and potential local adaptation to similar environmental conditions (Table S8, Fig. 3) (Messaoudi et et al. 2020). In contrast, the second clade comprises 16 strains originating from diverse geographical areas, including North America, North Africa, and the Mediterranean basin (Table S8). This clade showed greater genomic variability, as reflected by the diversity in accessory genes in the gene presence/absence matrix. The presence of strain-specific genes and variability in accessory gene content across regions suggests that geographic and ecological factors play a significant role in shaping the genomic architecture of *B. halotolerans* (Gong et al. 2023, Rafanomezantsoa et al. 2025).

Moreover, strain *B. halotolerans* F11 formed a distinct and stable branch within the second clade (Fig. 3), clustering with *B. halotolerans* OM-41, isolated from the Moroccan olive rhizosphere (Ajdig et al. 2025), and with strain *B. halotolerans* BCP32, isolated from deep-sea sediments in the Gulf of Naples, Italy. These results reflect the remarkable flexibility of *B. halotolerans* strains to adapt to diverse and extreme ecological niches, ranging from marine deep-sea environments to arid deserts and Saharan soils (Casella et al. 2025).

Furthermore, *B. halotolerans* F11 harbors 17 strain-specific genes (Table S9). Among these accessory genes, the fengycin core genes fenA and fenC were identified. This strain-specific gene pattern may be related to high sequence divergence compared with homologs in other *B. halotolerans* genomes, leading them to fall below the Roary detection threshold, or may also reflect true gene gain events acquired through horizontal gene transfer (Matthews et al. 2024).

### Genome mining for BGC prediction

**Figure 4:**
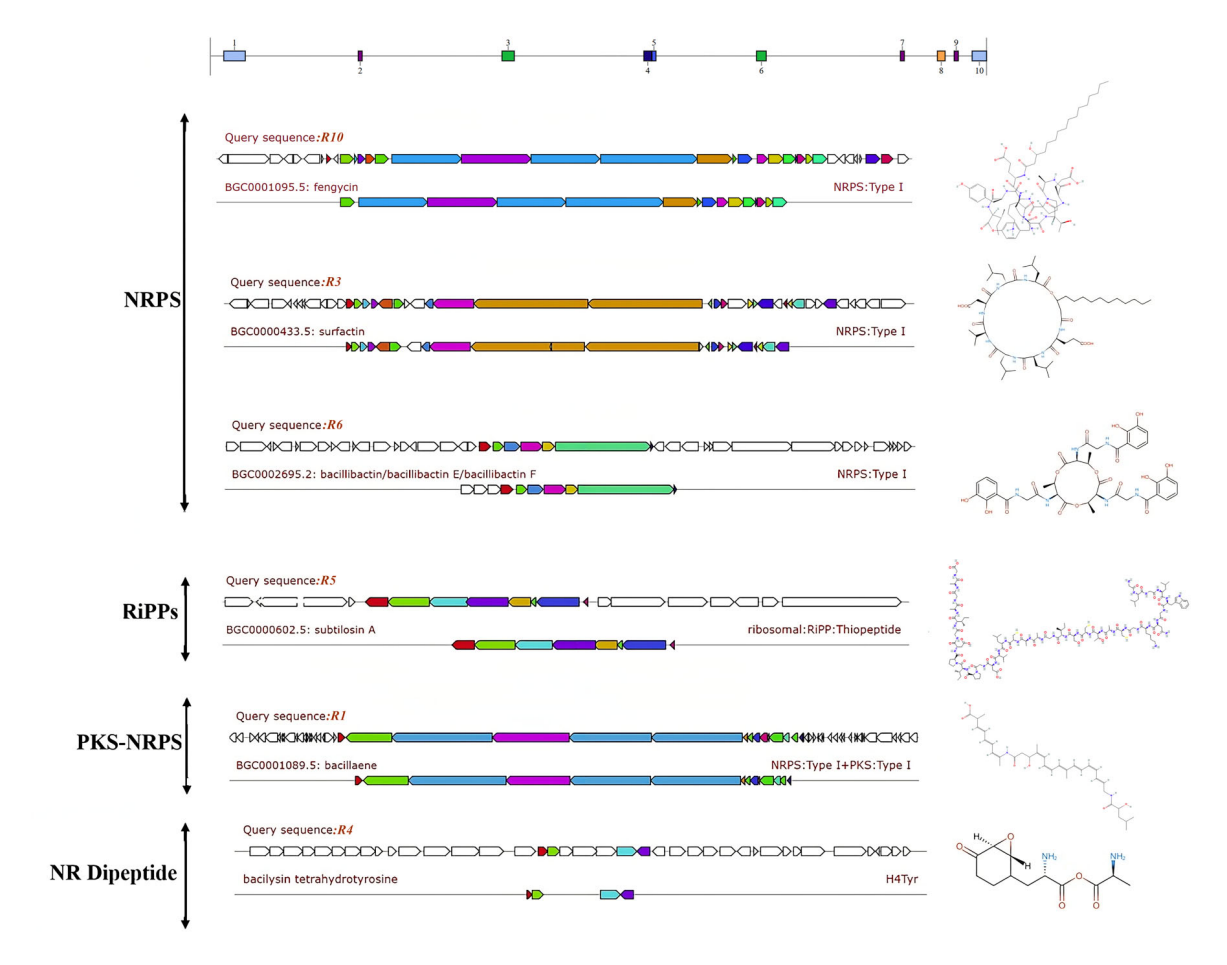
Genome annotation of *B. halotolerans* F11 using antiSMASH v8, showing the predicted BGCs and their corresponding core structures.

To perform an in-depth genome mining analysis of gene clusters involved in secondary metabolite biosynthesis, the *B. halotolerans* F11 genome was annotated using antiSMASH v8. The detected BGCs are summarized in Table 4 and Fig. 4.

**Table 4:** AntiSMASH annotation of the *B. halotolerans* F11 genome.

BGC positions in the F11 genome	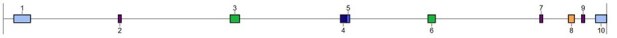				
	Biosynthetic classes	Closest known Metabolites	MIBiG accession	Regions	Similarity
R3	NRP	Surfactin	BGC0000433.5	1 561 167–1 626 565	86%
R6		Bacillibactin	BGC0000309.5	2 923 160–2 974 934	100%
R10		Fengycin	BGC0001095.5	4 076 816–4 154 202	80%
R1	Hybrid PK/NRP	Bacillaene	BGC0001089.5	71 907–187 006	100%
R5	RiPPs	Subtilosin A	BGC0000602.5	2 363 353–2 384 965	100%
R4	Dipeptide	Bacilysin	BGC0001184.4	2 320 258–2 361 676	100%
R8	T3PK	Unkown	–	3 890 991–3 932088	–
R2	Terpene	Unkown	–	791 556–812 362	–
R7		Unkown	–	3 693 054–3 713 944	–
R9		Unkown	–	3 980 866–4 002 764	–

NRP: nonribosomal peptide; PK–NRP: hybrid polyketide–non ribosomal peptide; RiPPs: ribosomally synthesized and posttranslationally modified peptides; T3PK : type 3 polyketide.

The online antiSMASH analysis predicted ten BGCs regions in *B. halotolerans* F11, covering a broad range of secondary metabolite classes (Fig. 4 and Table 4). These include three nonribosomal peptide (NRP) clusters encoding bacillibactin, surfactin, and fengycin. In addition, one hybrid polyketide–nonribosomal peptide (PK–NRP) cluster responsible for bacillaene biosynthesis and one dipeptide cluster encoding bacilysin were identified. Furthermore, one Ribosomally synthesized and Posttranslationally modified Peptides (RiPP) BGC corresponding to subtilosin A was detected (Table 4).

In line with the genomic analysis conducted by Steinke et al. (2021) across 310 complete genomes within the *B. subtilis* species complex, strain *B. halotolerans* F11 harbors several core secondary metabolite pathways shared with *B. subtilis* and closely related species. These include BGCs involved in the production of bacillibactin, surfactin, bacilysin, and bacillaene, which were identified as core clusters broadly conserved across the *B. subtilis* group. However, the fengycin cluster displays a clear clade-specific distribution and is predominantly associated with *B. velezensis* and *B. amyloliquefaciens* (Steinke et al. 2021).

Among the predicted BGCs, several displayed 100% similarity to well-characterized clusters, including those encoding bacillibactin (R6), bacillaene (region R1), subtilosin A (R5), and bacilysin (R4) (Table 4). In contrast, R10 (fengycin-like BGC) and R3 (surfactin-like BGC) showed high similarity to known clusters (80% and 86%, respectively) (Kai et al. 2025, Chakraborty et al. 2022). However, the remaining BGCs showed no similarity to any characterized clusters in the database (Table 4). This observation highlights an important limitation of the MIBiG database, which is based solely on currently characterized BGCs. Indeed, of the more than 36 454 microbial metabolites catalogued in NPAtlas (Poynton et al. 2025), only approximately 3015 have corresponding BGC entries in MIBiG (Zdouc et al. 2025). Therefore, the absence of matches for certain predicted metabolite clusters does not necessarily indicate novelty but may instead reflect incomplete database annotation (Khater et al. 2016).

### BiG-SCAPE analysis and evolutionary relationships

**Figure 5:**
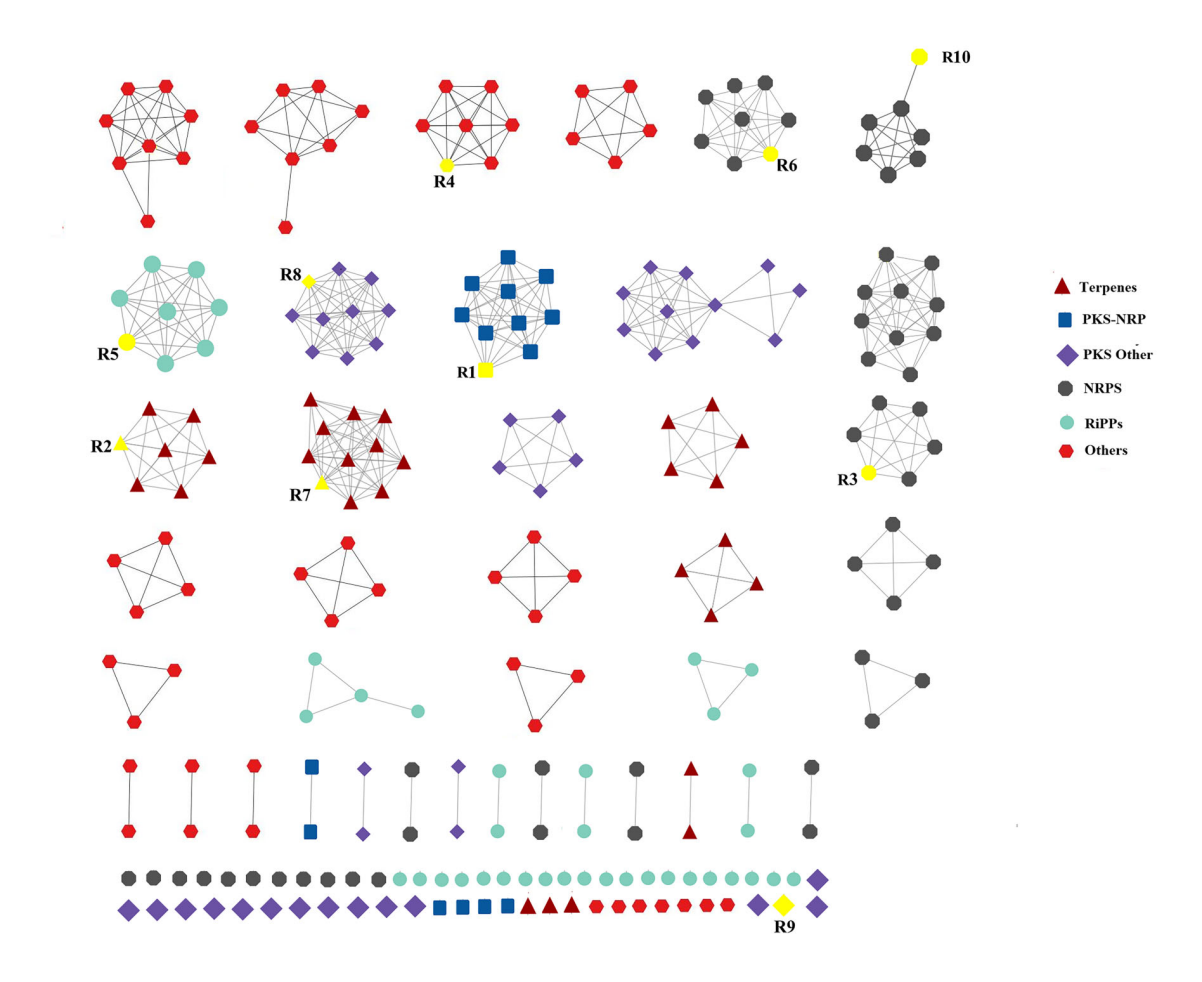
BiG-SCAPE sequence similarity networks of BGCs from various *Bacillus* species, showing the positions of BGC regions from strain F11, labeled with the letter “R” and shown as yellow nodes.

To elucidate the evolutionary relationships between the BGCs detected in *B. halotolerans* F11 through antiSMASH analysis and their closest homologs, a BiG-SCAPE analysis was conducted. For this purpose, sequence similarity networks were constructed using a dataset of 253 nonredundant BGCs derived from diverse *Bacillus* species (Table S10). The results are presented in Fig. 5.

A total of 253 BGCs were grouped into 100 distinct GCFs, connected by 462 edges including self-loops (Table S10). Among the 100 GCFs, 60 BGCs appeared as unique nodes (singletons) with no detectable homology at the 0.30 similarity cutoff (Fig. 5). The remaining 40 were identified as multimember GCFs, distributed across different biosynthetic classes: 10 NRPS, 6 RiPPs, 5 terpenes, 5 PKS, 2 hybrid PKS–NRPS as well as 12 other BGCs networks (Table S10).

The BGCs from *B. halotolerans* F11, highlighted in yellow, were distributed across 10 distinct GCFs, including one singleton (Fig. 5). Among them, R3 showed strong connections with surfactin BGCs identified in various *Bacillus* species, including the reference surfactin cluster (BGC0000433). These strong network connections indicate a close relationship and suggest potential similarity to canonical surfactin BGC. Interestingly, R10 annotated by antiSMASH as a fengycin-type BGC (Table 4), exhibited weak connectivity and appeared as an isolated node within the BiG-SCAPE network (Fig. 5), suggesting a potential divergence from well-characterized fengycin clusters.

### Analysis of fengycin BGCs in *B. halotolerans* F11 and related strains

Fengycin BGCs from *B. halotolerans* F11 and related strains were analyzed and compared using multiple bioinformatic tools. Clinker was used for comparative synteny analysis across fengycin clusters from different *B. halotolerans* strains, CORASON was applied to assess evolutionary divergence, and antiSMASH v8.0 was utilized to predict NRPS domain–module organization and substrate specificity.

#### Synteny analysis of fengycin BGCs across *B. halotolerans* strains

To assess whether the overall genomic organization of the R10 fengycin cluster from *B. halotolerans* F11 is conserved relative to well-characterized reference clusters, a synteny analysis was performed using clinker to evaluate gene order, orientation, and conservation (Gilchrist and Chooi 2021). For this purpose, the R10 cluster was compared with two canonical reference clusters representing plipastatin (BGC0000407) and fengycin-type (BGC0001095) BGCs. The results are shown in Fig. S4.

Comparative synteny analysis (Fig. S4) revealed that the core NRPS genes responsible for fengycin backbone biosynthesis are highly conserved across all examined clusters, maintaining the canonical fenA–fenB–fenC–fenD–fenE organization (Shu et al. 2002). Therefore, the R10 cluster corresponds to a complete fengycin BGC (Table S11 and Fig. S5) and displays a conserved genomic architecture consistent with canonical fengycin clusters reported in several *Bacillus* species (Wu et al. 2007, Yin et al. 2024).

These findings were further supported by a domain-level comparative analysis based on the Pfam domain composition of GCFs related to fengycin-type BGCs, as identified through the BiG-SCAPE analysis (Fig. 5). The analysis was performed using CORASON (Navarro-Muñoz et al. 2020). This approach enabled the systematic comparison of the fengycin BGC from *B. halotolerans* F11 with fengycin-type clusters identified across different *Bacillus* species, thereby providing a refined view of their structural conservation and evolutionary relationships.

The resulting phylogenetic tree (Fig. S6) confirmed the strong conservation of the core fengycin biosynthetic framework across all clusters, supporting the evolutionary stability of the enzymatic machinery responsible for fengycin decapeptide biosynthesis across *Bacillus* species.

**Figure 6:**
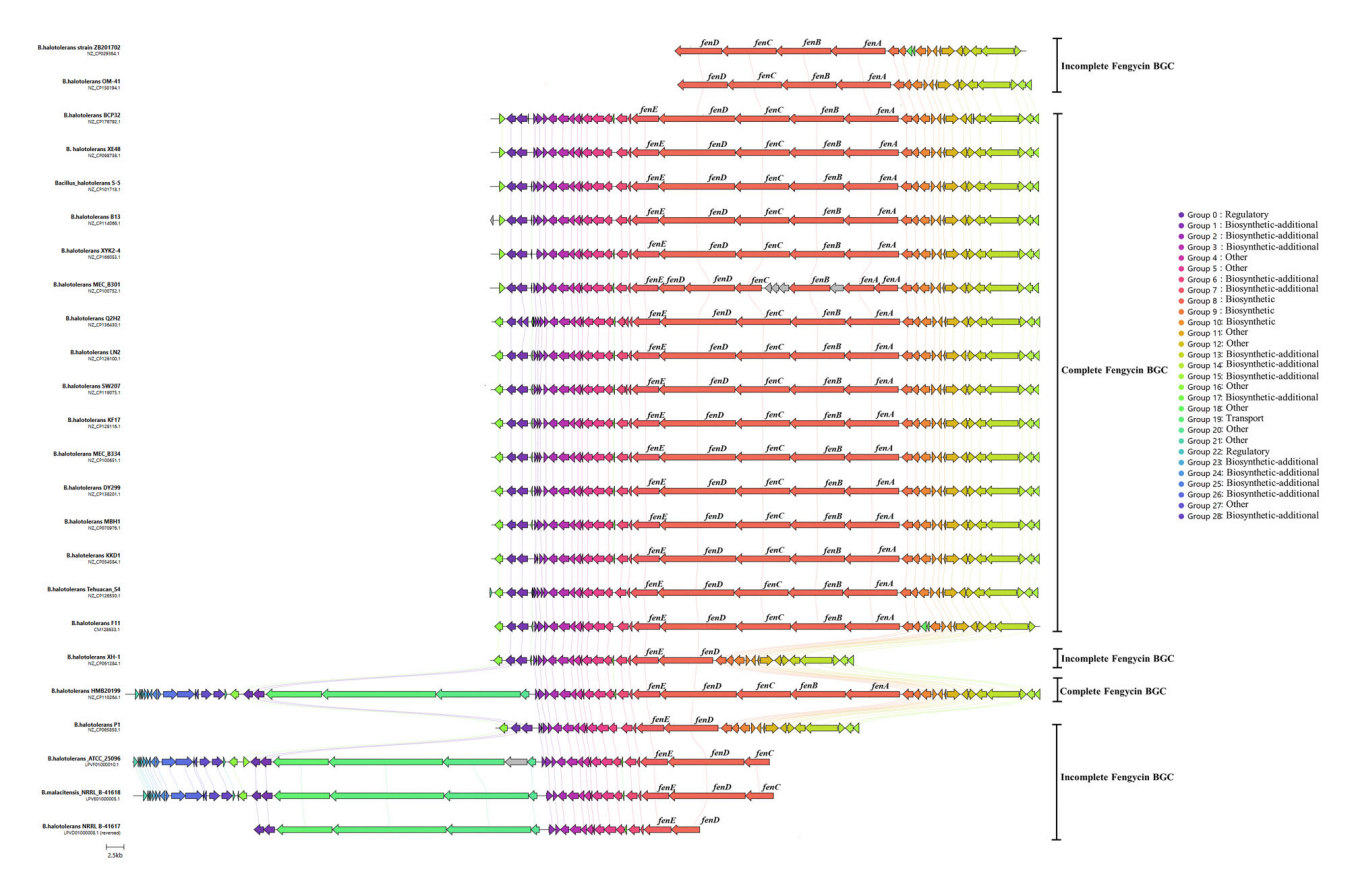
Comparative synteny analysis of fengycin BGCs across 24 *B. halotolerans* strains

Furthermore, to expand the previous synteny results, we investigated patterns across all complete genomes of the *B.halotolerans* group to dissect the intraspecies diversity of fengycin BGCs. For this purpose, a comparative synteny analysis was conducted using 24 fengycin BGCs from different *B. halotolerans* strains. The results are presented in Fig. 6.

The results (Fig. 6) showed that the fengycin BGC is a conserved feature representing an intraspecies pattern characteristic of *B. halotolerans*, as it was detected in all 24 analysed genomes. Among them, 17 harbored a complete canonical fengycin cluster (fenA–fenE), including the fengycin cluster of strain *B. halotolerans* F11, which showed a highly conserved gene order and orientation, indicating strong evolutionary stability and suggesting a shared ancestral origin within *B. halotolerans* (Zeng et al. 2021). In contrast, strain *B. halotolerans* OM41, despite their close evolutionary relationship to *B. halotolerans* F11 (Fig. 6), harbored incomplete fengycin BGCs lacking one core biosynthetic gene (*fenE*). However, other strains exhibited a more pronounced reduction of the cluster, lacking more than two core genes (Fig. 6).

Similar clade-linked patterns of BGC distribution have been reported in the *Bacillus* genus, where specific CLPs clusters such as fengycin, plipastatin, or lichenysin follow phylogenetic boundaries. Therefore, the conserved occurrence of fengycin within *B. halotolerans* likely reflects vertical inheritance from a common ancestor and highlights its potential ecological and functional relevance within this species (Dunlap et al. 2020, Steinke et al. 2021).

Interestingly, a marked divergence from the canonical fengycin BGC architecture was observed in *B. halotolerans* strain HMB20199. This strain harbors the complete set of fengycin core genes (fenA–fenE), together with four additional large NRPS genes showing strong homology to those typically found in iturin-family lipopeptide clusters (ituA–ituD/bamA–bamD) (Dunlap et al. 2019). Other *B. halotolerans* strains, including ATCC 25096, NRRL B-41617, and NRRL B-41618, exhibit a similar mosaic organization. However, in these strains the clusters are incomplete and lack two or more core fengycin biosynthetic genes, suggesting partial cluster degeneration (Fig. 6).

**Figure 7:**
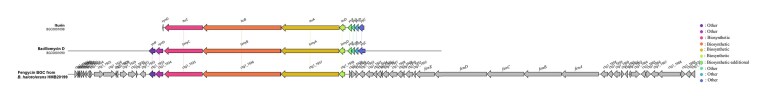
Comparative synteny analysis of the fengycin BGC from *B. halotolerans* HMB20199 and two iturin-family BGCs (iturin and bacillomycin D).

To further characterize this atypical organization, a comparative synteny analysis was performed between the fengycin BGC of *B. halotolerans* HMB20199 and two representative iturin-family BGCs, including iturin and bacillomycin D. The results are presented in Fig. 7.

The results showed a strong collinearity between the additional NRPS genes of HMB20199 and known iturin-family clusters (Fig. 7), indicating a close structural relationship. These findings suggest that the presence, within the same BGC, of canonical fengycin core genes together with iturin-like genes likely results from recombination events, possibly involving horizontal gene transfer within the *Bacillus* lipopeptide superfamily (Zotchev, 2026).

Furthermore, genome analysis of other *Bacillus* species harboring fengycin BGCs with mosaic organization revealed that *B. tequilensis* ATCC BAA-819^T^, isolated from a 2000-year-old archaeological site in Mexico, as well as *B. inaquosorum* KCTC 13429^T^, possess BGCs with comparable organization. This observation suggests that the occurrence of hybrid fengycin–iturin clusters, even in phylogenetically distinct or historically isolated lineages, indicates that such hybrid BGC architectures are not recent artifacts but may instead represent long-standing evolutionary arrangements (Gatson et al. 2006, Chouaia et al. 2024).

#### Analysis of fengycin NRPS domain and module organization

**Figure 8:**
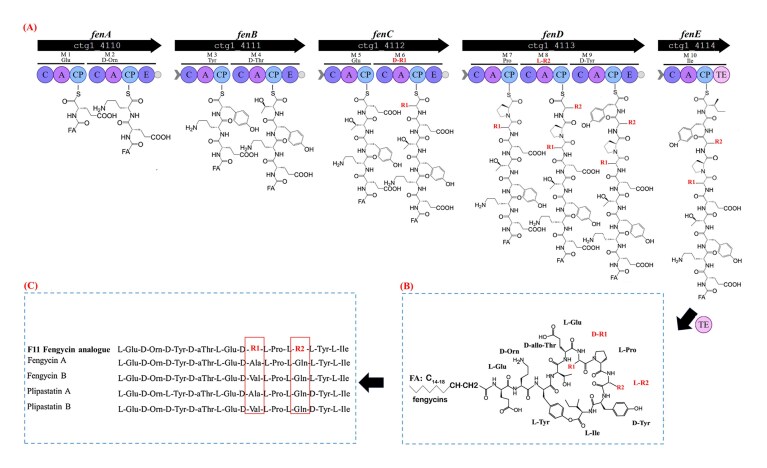
Predicted fengycin NRPS modular organization and proposed biosynthetic mechanism of the fengycin analogue from *B. halotolerans* F11. (A) Peptide chain elongation catalysed by the NRPS megaenzyme, illustrating the incorporation of two unknown amino acid residues at modules M6 and M8. The fatty acid chain is incorporated by the starter condensation domain of FenA prior to initiation of module 1 (M1). (B) Proposed structure of the *B. halotolerans* F11fengycin analogue, showing variable amino acid residues (R1 and R2) and an unknown fatty acid chain length. (C) Structural diversity within the fengycin family compared with the proposed fengycin analogue from *B. halotolerans* F11.

**Figure 9:**
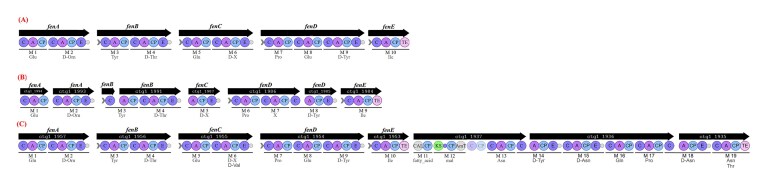
Predicted NRPS megaenzyme organization encoded by the BGCs identified in different *B. halotolerans* strains. (A) NRPS modular organization and substrate specificity predicted for 12 *B. halotolerans* strains: XE48, Q2H2, S-5, B13, LN2, SW207, KF17, MEC_B334, DY299, MBH1, KKD1, and Tehuacan_S4. (B) NRPS modular organization and substrate specificity predicted for strain *B. halotolerans* MEC_B301. (C) NRPS modular organization and substrate specificity predicted for strain *B. halotolerans* HMB20199, showing mosaic hybridization between fengycin and iturin-family lipopeptide modules.

To further explore the divergence observed in fengycin BGCs at the enzymatic level, a detailed analysis of the modular and domain organization of the NRPS assembly line was performed using antiSMASH v8.0. The results are presented in Figs 8 and 9.

For the fengycin BGC of strain *B. halotolerans* F11, the analysis revealed that although the overall 10-module NRPS architecture of the fengycin assembly line is consistent with the canonical fengycin NRPS megaenzyme (Zhao et al. 2013), specific variations were observed in the substrate specificity of certain adenylation (A) domains, particularly in modules M6 and M8, encoded by *fenC* and *fenD*, respectively (Fig. 8A). In canonical fengycin biosynthesis, M6 incorporates d-Ala or d-Val, whereas M8 incorporates Gln into the decapeptide backbone (Yin et al. 2024) (Fig. 8A). However, in *B. halotolerans* F11, antiSMASH v8.0 predictions for these modules showed ambiguous substrate assignments (Fig. 8B), suggesting potential incorporation of alternative amino acid residues (Fig. 7C).

When compared with the predicted fengycin NRPS megaenzymes of other *B. halotolerans* strains harboring complete fengycin BGCs, only two strains were found to possess adenylation (A) domains in modules M6 and M8 with substrate specificities similar to those predicted for *B. halotolerans* F11. These strains are *B. halotolerans* BCP32, the closest relative of *B. halotolerans* F11 based on core-genome phylogenetic analysis (Fig. 3), and *B. halotolerans* XYK2, isolated from rhizosphere soil in Shaanxi Province, China (Table S8). In contrast, the twelve other *B. halotolerans* strains showed variation in substrate specificity in module M6 only (Fig. 9A), which represents a key determinant of fengycin structural diversity (Vanittanakom et al. 1986).

Furthermore, the predicted fengycin NRPS modular organization in *B. halotolerans* MEC_B301 revealed only nine modules, suggesting the incorporation of nine amino acid residues instead of the 10 residues characteristic of the canonical fengycin decapeptide (Fig. 9B). This may indicate possible structural divergence compared to reference fengycin compounds. Moreover, NRPS domain analysis of the fengycin BGCs from *B. halotolerans* HMB20199, *B. inaquosorum* KCTC 13429^T^, and *B. tequilensis* ATCC BAA-819^T^ revealed an extended NRPS assembly line comprising 19 modules, potentially capable of incorporating up to 17 amino acid residues (Fig. 9C). This observation is consistent with the mosaic organization identified at the genomic level (Fig. 6). However, experimental validation through purification and structural elucidation is required to determine if these mosaic BGCs are functionally expressed and lead to the production of novel lipopeptide scaffolds combining structural features of both the fengycin and iturin families.

These genomic findings indicate that strains belonging to the *B. halotolerans* species can be considered a promising genomic reservoir of novel NRPS-derived lipopeptides, including potentially new fengycin analogues. This biosynthetic versatility underscores their relevance for future exploration in NP discovery programs.

### Metabolomic characterization of *B. halotolerans* F11 strain

#### OSMAC-based modulation of secondary metabolite production

A medium-dependent antimicrobial screening was performed according to the One Strain–Many Compounds (OSMAC) strategy prior to metabolomic analysis of *B. halotolerans* F11. This approach enables the identification of culture conditions that maximize antimicrobial activity, thereby ensuring that metabolomic profiling reflects the biosynthetic pathways responsible for the observed antimicrobial phenotype (Hewage et al. 2014). Therefore, strain *B. halotolerans* F11 was cultivated in five different culture media to evaluate the effect of growth conditions on its antimicrobial potential. The results are summarized in Table S12.

Under the OSMAC approach, growth of *B.halotolerans* F11 on SM and ISP2 media resulted in antimicrobial activity with inhibition zones ranging from 8 to 30 mm, demonstrating activity against a broad range of tested microorganisms. Compared with the antimicrobial profile obtained on TSA medium (Table 1), the OSMAC-based screening revealed an expansion of the activity spectrum, as several previously resistant microorganisms, including *E. coli, S. aureus*, and the yeast *C. albicans*, became susceptible when *B. halotolerans* F11 was grown on SM and ISP2 media (Table S12). Notably, a marked enhancement of antimicrobial activity was observed against the Gram-positive bacteria *S. aureus* and both *C. albicans* strains (10 and 26) when *B. halotolerans* F11 was grown on ISP2 and SM media, compared with TSA medium.

The medium-dependent shifts in antimicrobial activity observed for *B. halotolerans* F11 during OSMAC-based screening highlight the strong influence of culture conditions, particularly carbon and nitrogen availability, on secondary metabolite production (Zheng et al. 2025). These conditions can directly activate silent or weakly expressed BGCs, leading to the synthesis of bioactive metabolites that are not produced under initial culture conditions and that contribute to the increased antimicrobial activity observed (Table S12).

Based on the OSMAC screening, ISP2 was selected for MIC evaluation using the 96-well plate method, following growth of *B. halotolerans* F11 in ISP2 broth. The results are presented in Table S13.

The MIC results confirmed the OSMAC findings, demonstrating significant antimicrobial activity, with a lowest MIC value of 1.6 µg/ml against most tested microorganisms, except for the three Gram-negative bacteria *K. pneumoniae, E. coli*, and *P. aeruginosa* (Table S12).

Moreover, discrepancies in activity against *Y. enterocolitica* and *P. aeruginosa* were observed between solid ISP2 medium (OSMAC assay) and liquid ISP2 broth (MIC determination). This variation is most likely attributable to differences in culture conditions. Several studies have reported that the expression of BGCs can be strongly influenced by factors such as growth state (solid versus liquid), oxygen availability, and quorum-sensing regulation, all of which may significantly affect metabolite production profiles. These differences likely explain the observed variation in antimicrobial activity (Bode et al. 2002).

Based on these results, the untargeted metabolomic profiling was conducted under the same culture conditions used for the MIC assays to directly correlate the antimicrobial phenotype with the secondary metabolite profile and genomic features of *B. halotolerans* F11.

#### Untargeted metabolomic analysis of *B. halotolerans* F11 strain

**Figure 10:**
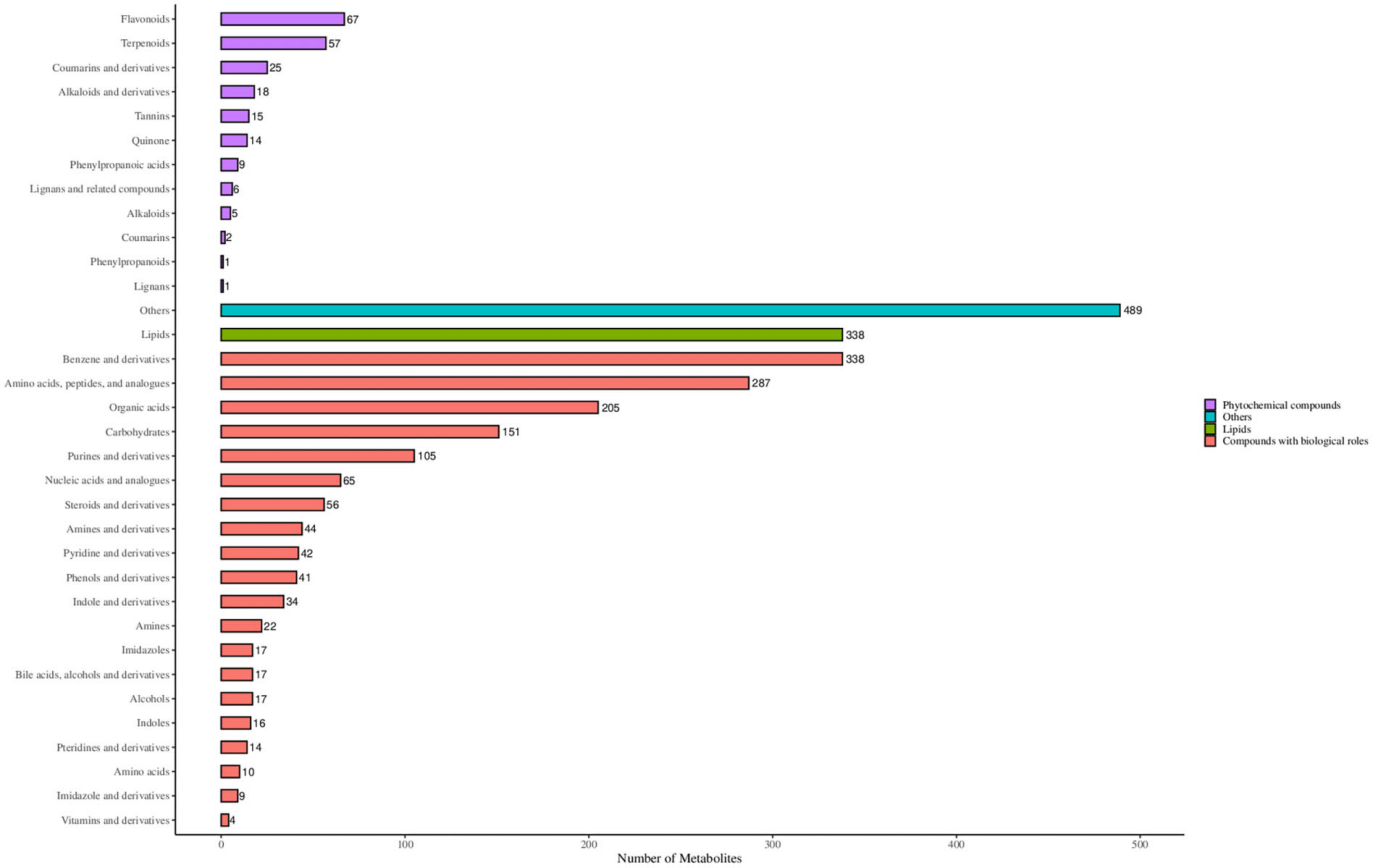
Bar chart illustrating the distribution of metabolites identified in the untargeted metabolomic profile of *B. halotolerans* F11. The *x*-axis represents the number of detected metabolites, while the *y*-axis indicates their metabolic classifications.

Untargeted metabolomic analysis was performed, after culturing the strain *B. halotolerans* F11 in liquid ISP2 medium and incubating at 30°C with shaking at 150 rpm, to obtain a comprehensive overview of the metabolite profile of strain *B. halotolerans* F11 under the same culture conditions used for MIC determination (Table S13). The identified metabolites and their distribution across major chemical classes are presented in Fig. 10.

The untargeted metabolomic analysis of the fermentation broth of strain *B. halotolerans* F11 revealed a total of 9719 metabolites, of which 3453 were identified (Data S2). Among the identified metabolites, lipid-derived compounds and benzene derivatives represented the largest class, each accounting for 13.3%, followed by amino acids and peptides (11.29%), organic acids (8.07%), and carbohydrates (5.94%) (Fig. 10). This distribution highlights the broad metabolic versatility of *B. halotolerans* F11 under the tested conditions. Notably, this metabolic pattern differs from that reported for *B. velezensis* B115 cultivated in LB medium at 28°C, where amino acids were the predominant class (24.81%) among 2318 detected metabolites (Chen et al. 2025).

Interestingly, several phytochemical-like compounds, such as flavonoids, terpenoids, and coumarins, were also detected (Fig. 10) (Yahla et al. 2025). Although these molecules are not typically synthesized de novo by *B. halotolerans* F11 strain, their presence may be attributed to the ISP2 culture medium, which contains plant-derived ingredients such as malt extracts. Nevertheless, it remains plausible that strain *B. halotolerans* F11 contributes to their structural diversification through biotransformation, converting medium-derived substrates into novel metabolites (Messaoudi et et al. 2022b).

**Figure 11:**
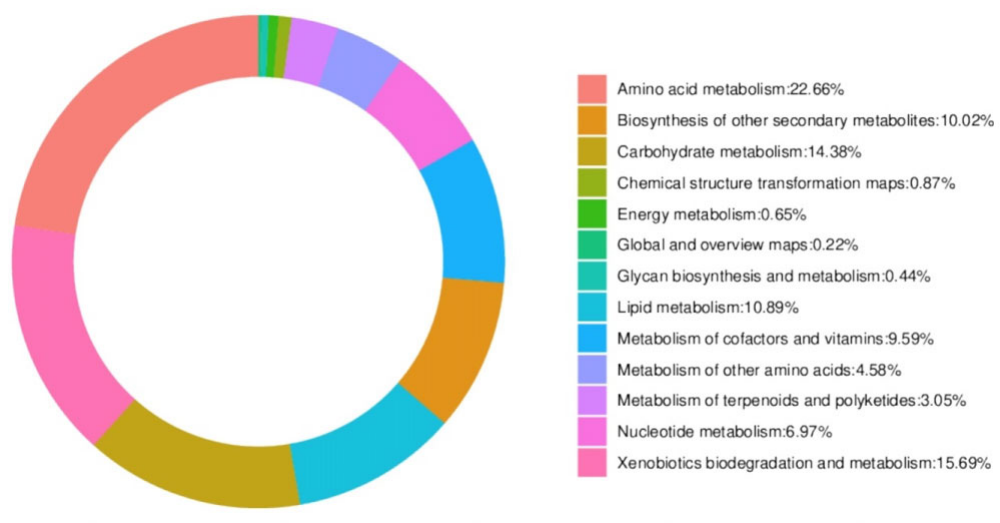
Donut chart showing the distribution of metabolites across KEGG pathways. Colors represent different metabolic categories, and percentages indicate the proportion of metabolites within each pathway relative to the total annotated metabolites.

To gain deeper insight into their biological significance, the detected metabolites were mapped to biochemical pathways using KEGG annotations, based on their super pathway classification. This approach provides a comprehensive picture of the metabolic activities of *B. halotolerans* F11 strain under the given culture conditions and allowed us to link the metabolite profile of *B. halotolerans* F11 to specific metabolic routes that may contribute to its observed antimicrobial activity (Janßen et al. 2014). The obtained results are presented in Fig. 11.

The KEGG pathway classification of metabolites detected in *B.halotolerans* F11 cultured in ISP2 broth at 30°C and 150 rpm revealed a broad distribution across both primary and secondary metabolic pathways (Fig. 11). Within primary metabolism, amino acid metabolism accounted for the largest proportion (22.66%), followed by carbohydrate (14.38%) and lipid metabolism (10.89%). Secondary metabolism was also well represented (10.02%), including pathways related to terpene and polyketide biosynthesis (3.05%). Such distribution highlights the close interdependence between primary and secondary pathways, where primary metabolites provide essential precursors and energy required for secondary biosynthetic processes (West et al. 2023).

Metabolomic profiling of *B. halotolerans* F11 further supports this metabolic orientation (Data S2). Several acyl-CoA derivatives were detected, including acetyl-CoA, methylmalonyl-CoA, and ethylmalonyl-CoA, which are key intermediates in fatty acid (FA) metabolism (Janßen et al. 2014). In addition, multiple metabolites associated with amino acid metabolism were identified (Data S2), indicating active biosynthetic pathways involved in the production of various amino acid precursors. These findings demonstrate that FA and amino acid metabolic routes are highly active under the tested culture conditions, generating essential starter and extender units that serve as precursors for the biosynthesis of polyketide- and lipopeptide-derived compounds (Li et al. 2023).

### Genomic–metabolomic correlation and putative biosynthetic pathways

To establish a link between the secondary metabolites produced by strain *B. halotolerans* F11 and its predicted BGCs, metabolomic data were integrated with the genomic annotation of the strain *B. halotolerans* F11. This combined analysis provided deeper insight into which BGCs of *B. halotolerans* F11 are upregulated when cultured in ISP2 broth at 30°C and 150 rpm for 5 days, thereby contributing to the observed antimicrobial activity (Valenzuela et al. 2024).

Metabolomic analysis revealed that, among the ten antiSMASH-predicted BGCs in *B. halotolerans* F11 (Table 4), two BGCs were directly correlated with their corresponding metabolites. These compounds were detected at retention times of 6.825, and 7.431 min, exhibiting molecular ion clusters of [M+H]⁺ at m/z 883.608 and 581.412, with molecular formulas C₃₉H₄₃N₆O_18_ and C₃₄H₄₉N₂O₆, respectively (Data S2). Database searches against Antibase and the Dictionary of Natural Products identified these metabolites as bacillibactin and bacillaene, respectively.

Both bacillibactin and bacillaene are well known for their broad-spectrum antimicrobial activity. Bacillaene is a linear polyketide produced via a type I PKS pathway that exhibits antimicrobial properties against a range of bacterial competitors (Miao et al. 2023), while bacillibactin acts as a siderophore that sequesters iron and thereby deprives pathogens of this essential nutrient, inhibiting their growth (Sakajoh et al. 1987, Chakraborty et al. 2022). Therefore, part of the antibacterial activity observed for *B. halotolerans* F11 (Table 1) can be attributed to these bioactive metabolites.

In contrast, BGCs associated with the biosynthesis of bacilysin, fengycin, surfactin, and subtilosin A were clearly identified at the genomic level; however, their corresponding metabolites were not detected in the metabolomic dataset (Data S2). Among these, the bacilysin BGC identified in *B. halotolerans* F11 appears incomplete when compared with the canonical bacilysin biosynthetic cluster, which likely explains the absence of the corresponding metabolite. On the other hand, the lack of detection of fengycin, surfactin, and subtilosin A is more likely attributable to methodological constraints (Lee et al. 2025). Specifically, the UPLC–HRMS/MS acquisition method used in this study was optimized for the detection of low-molecular-weight compounds (<1000 Da), thereby limiting the detection of larger peptides such as fengycin (MW 1460–1530 Da), surfactin (MW 1008–1050 Da), and subtilosin A (MW ≈ 3399 Da). Nevertheless, although their presence was not experimentally validated under the current analytical setup, the metabolomic profile of *B. halotolerans* F11 still suggests an active biosynthetic state consistent with lipopeptide- and peptide-associated metabolic pathways under the applied culture conditions (Fig. 11).

**Figure 12:**
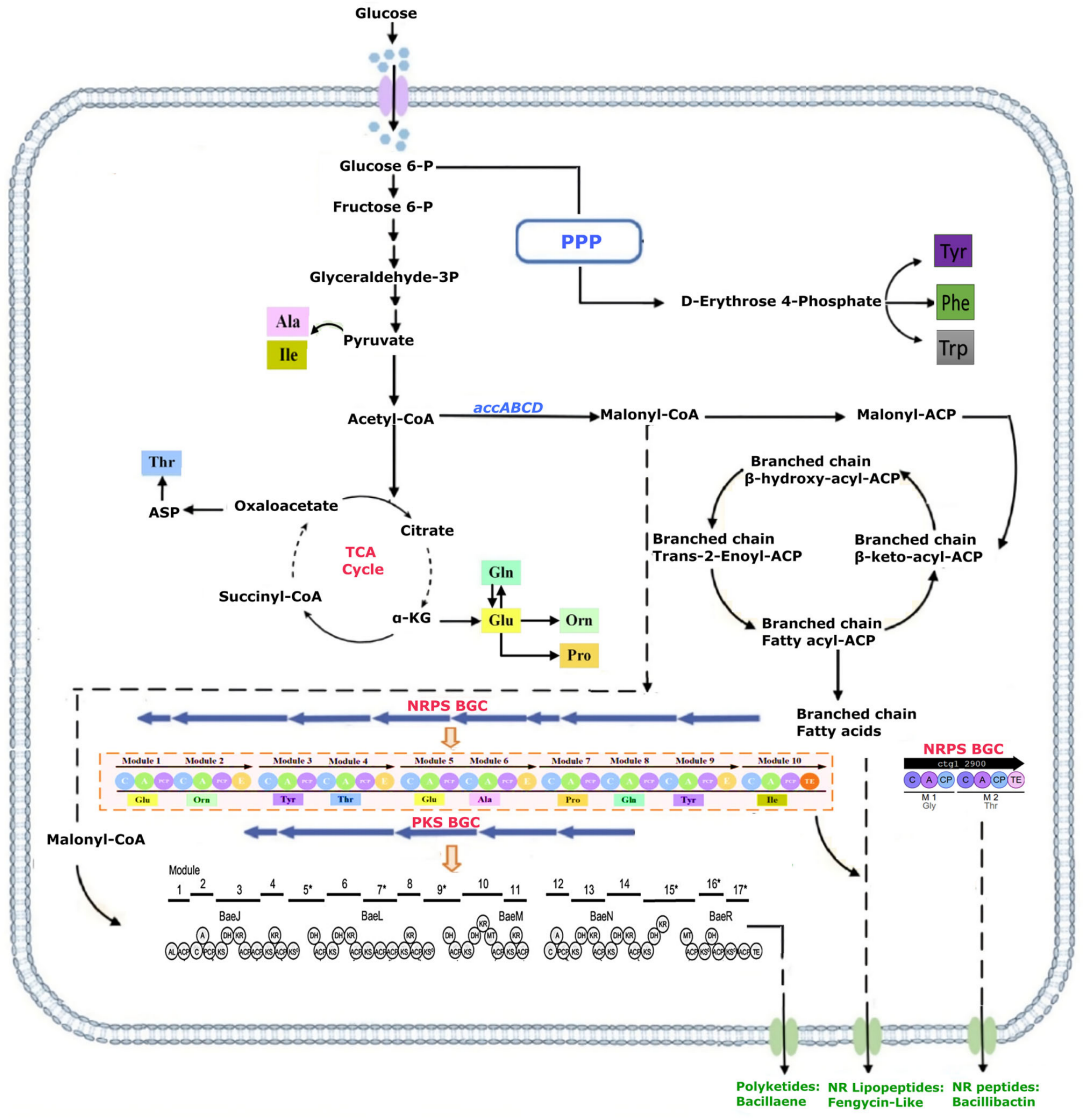
Proposed and speculative metabolic pathways involved in secondary metabolite biosynthesis in *B. halotolerans* F11 after 5 days of incubation in ISP2 medium at 30°C under shaking at 150 rpm. PPP: pentose phosphate pathway; TCA: tricarboxylic acid cycle; AccABCD: acetyl-CoA carboxylase complex; α-KG: α-ketoglutarate; NRPS: nonribosomal peptide synthetase; BGC: biosynthetic gene cluster; NR: nonribosomal; and PKS: polyketide synthase.

Building on the integration of metabolomic profiling (Data S2) with annotated secondary metabolites (Fig. 4) and predicted BGCs (Table 4) a putative reconstruction of the major secondary metabolite biosynthetic pathways in *B. halotolerans* F11 was proposed (Fig. 12).

Figure 12 presents a speculative model derived from untargeted metabolomics data and genome-based BGC predictions, illustrating the main biosynthetic pathways involved in the production of the polyketide bacillaene, the nonribosomal peptides bacillibactin and the fengycin-like compounds in *B. halotolerans* F11. As such, it provides a conceptual overview of potential metabolic linkages and biosynthetic relationships rather than a confirmed biochemical pathway.

According to this model (Fig. 12), the process begins with the utilization of carbohydrates present in the ISP2 medium. Strain F11 primarily metabolizes glucose, supplied at 10 g/l in the medium. This carbon and energy source is channelled into central metabolic pathways, including glycolysis, the pentose phosphate pathway, and the tricarboxylic acid cycle, generating key intermediates such as pyruvate, acetyl-CoA, d-erythrose-4-phosphate, oxaloacetate, and α-ketoglutarate. These metabolites serve as essential precursors for the biosynthesis of FAs and amino acids.

Furthermore, FA metabolism begins with acetyl-CoA, which is converted into malonyl-CoA via the acetyl-CoA carboxylase complex (AccABCD), a metabolite also detected in the metabolomic profile of strain F11. Malonyl-CoA is subsequently transformed into malonyl-ACP, which initiates FA chain elongation and also serves as an extender unit for polyketide biosynthesis, such as in the production of bacillaene (Fig. 12).

In parallel, amino acids are synthesized from central metabolic intermediates. Alanine and isoleucine derive from pyruvate, aromatic amino acids (tyrosine, phenylalanine, and threonine) originate from d-erythrose-4-phosphate, while glutamate, glutamine, and asparagine are produced from oxaloacetate and α-ketoglutarate (Fig. 12). These amino acids represent essential monomers for the biosynthesis of NRPs, including bacillibactin and fengycin-like compounds.

The biosynthetic enzymes, PKSs and NRPSs, catalyze the assembly of secondary metabolites (Fig. 12). These megaenzymes activate and incorporate building blocks such as acyl-CoA derivatives and amino acid monomers to construct bacillaene, bacillibactin, and fengycin-like lipopeptides. The resulting compounds may subsequently undergo cyclization and other postsynthetic modifications before being secreted into the extracellular environment.

## Conclusion

This study was driven by the global challenge of antimicrobial resistance and aimed to explore the genome of the Algerian soil isolate F11 and related *B. halotolerans* strains to identify divergent BGCs potentially encoding uncharacterized CLPs. Our results show that strain *B. halotolerans* F11 carries a divergent fengycin BGC, while *B. halotolerans* HMB20199 harbors a mosaic fengycin–iturin hybrid cluster. These findings highlight members of the species *B. halotolerans* as promising genomic reservoirs of NRPS-derived lipopeptides with potential antimicrobial relevance. Nevertheless, additional *in vitro* investigations, including extraction, purification, and structural elucidation, are required to validate these predictions and confirm the expression of the identified fengycin biosynthetic genes.

## Funding

None declared.

## Supplementary Material

xtag037_Supplemental_Files
