# Supplementary material for: Comparative genome mining and metabolomics reveal divergent NRPS-derived fengycin biosynthetic gene clusters in Bacillus halotolerans
Source: FEMS Microbes. 2026 Jun 22;7:xtag037. doi: 10.1093/femsmc/xtag037 (PMC13322031; doi:10.1093/femsmc/xtag037)
Supplement: xtag037_Supplemental_Files [file xtag037_supplemental_files.zip › Supplementary_material.docx]

**Comparative Genome Mining and Metabolomics Reveal Divergent NRPS-Derived Fengycin Biosynthetic Gene Clusters in *Bacillus halotolerans***

Mokhtar Salah Eddine BELHADJ,**^1,2^** Omar MESSAOUDI,**^3,4,5*^** Mohamed YOUSFI,**^2^** Amina BAKHROUF**^1^**

**^1^** Laboratory of Analysis, Treatment and Valorization of Pollutants of the Environmental and Products, Faculty of Pharmacy, University of Monastir, Monastir, Tunisia

**^2^** Laboratoire des Sciences Fondamentales (LSF), Université Amar Telidji de Laghouat, Route de Ghardaïa, BP 37G, Laghouat, 03000, Laghouat, Algeria

**^3^** Research Unit of Medicinal Plant (RUMP) Attached to Center of Biotechnology (CRBt, 3000, Constantine), Laghouat 03000, Algeria

**^4^** Department of Biology, Faculty of Science, University of Amar Telidji, Laghouat 03000, Algeria

**^5^** Laboratory of Applied Microbiology in Food and Environment, Abou Bekr Belkaïd University, Tlemcen 13000, Algeria

*** Corresponding author.** *E-mail address*: [o.messaoudi@lagh-univ.dz](mailto:o.messaoudi@lagh-univ.dz) (O. Messaoudi). **ORCD**: <https://orcid.org/0000-0002-5684-294X>

**Supplementary Methods S1. Isolation of Spore-Forming Bacteria.**

The thermal shock method was used to selectively isolate spore-forming bacteria from various soil samples collected from different Algerian Sahara regions, following the protocol described by **Al-Humam, 2016.** Briefly, four grams of soil sample were suspended in sterile water, heat-treated at 60 °C for 1 h, and left to settle at room temperature for 2 h. Subsequently, an aliquot of 0.1 mL from each treated suspension was spread onto nutrient agar plates. The plates were incubated at 37 °C for five days and monitored daily for bacterial growth**.** Morphologically distinct colonies were selected, and pure isolates were obtained through repeated subculturing. The purified strains were stored at –20 °C in tryptic soy broth (TSB) supplemented with 30% glycerol, and preserved at –80 °C for long-term storage **(Kumar et al.,2021, Pawar et al.,2018).**

**Table S1.** Locations and soil types of sampling sites

| Sampling sites | Location | Type of Environment |
| --- | --- | --- |
| kaf el melh (Laghouat) | 33°38′47.120″ N   1°51′6.720″ E | Salt Mountain |
| Kef mokrane (Laghouat) | 33°48′00″N 2°51′54″E﻿ / ﻿33.8°N 2.865°E | Sand |
| Water taken from a cooling tower –Air Separation Unit | 33°48′00″N 2°51′54″E﻿ / ﻿33.8°N 2.865°E | Industrial Effluents |
| Oued M’zi (Laghouat) | 33°48′00″N 2°51′54″E﻿ / ﻿33.8°N 2.865°E | Soil |

**Table S2.** Culture media compositions used in this study

| **Nutrient Agar (NA)** | **ISP2** | **Methylophila Medium (YIM38)** | **Soybean Medium (SM)** | **Tryptic soy agar (TSA)** | **Mueller–Hinton agar (MH)** |
| --- | --- | --- | --- | --- | --- |
| - Meat extract: 1.0 g/L. - Yeast extract: 2.5 g/L. - Peptone: 5.0 g/L. - NaCl: 5.0 g/L. - Agar-agar: 15.0 g/L. - Distilled Water: 1 L. - pH 7.3. | - Yeast extract: 4 g/L. - Malt extract: 10 g/L. - Glucose: 4 g/L. - Agar: 20 g/L. - Distilled water: 1 L. - pH 7.3. | - Yeast Extract: 3 g/L - Malt Extract: 3 g/L. - Glucose: 10 g/L. - Peptone: 5 g/L; - MgSO₄·7H₂O: 0.5 g/L - NaCl: 0.5 g/L - CaCO₃: 2 g/L - Agar-agar: 15.0 g/L. - Distilled Water: 1 L. - pH 7.3. | - Soybean: 20 g/L. - Mannitol: 20 g/L - Glucose: 4 g/L - Agar-agar: 15.0 g/L. - Distilled Water: 1 L. - pH 7.3. | - Pancreatic digest of casein: 15 g/L. - Peptic digest of soybean 5 g/L. - NaCl: 5 g/L. - Agar-agar: 15.0 g/L. - Distilled Water: 1 L.   pH 7.3. | - Beef extract: 20g/L. - Casein hydrolysate: 17.5 g/L - Starch: 15 g - Agar: 17 g/L. - Distilled water: 1 L. - pH 7.3. |

**Table S3.** Main characteristics of the five selected aerobic spore-forming bacterial strains.

| Characters | F11 | F12 | F2 | F8 | F9 |
| --- | --- | --- | --- | --- | --- |
| Gram strain | Positive | Positive | Positive | Positive | Positive |
| Cell shape | rod-shaped | rod-shaped | Rod-shaped | Rod-shaped | Rod-shaped |
| Ability of spore formation | yes | yes | yes | yes | yes |
| Catalase | + | + | + | + | + |
| Nitrate reduction | + | + | + | + | + |
| Starch hydrolysis | + | + | + | + | + |
| Gelatin hydrolysis | + | + | + | + | + |

**Table S4.** Biochemical profiles of the selected aerobic spore-forming bacterial strains using API 50CHB and API 20E.

| API 50CHB | Biochemical tests | F11 | F12 | F2 | F8 | F9 |
| --- | --- | --- | --- | --- | --- | --- |
| 0 | Control | - | - | - | - | - |
| 1 | GLY | + | + | + | + | + |
| 2 | ERY | + | + | - | - | - |
| 3 | DARA | + | + | + | + | + |
| 4 | LARA | + | + | + | + | + |
| 5 | RIB | + | + | + | + | + |
| 6 | DXYL | + | + | + | + | + |
| 7 | LXYL | + | - | + | + | + |
| 8 | ADO | + | - | + | + | + |
| 9 | MDX | - | + | + | + | + |
| 10 | GAL | + | + | + | + | + |
| 11 | GLU | + | + | + | + | + |
| 12 | FRU | + | + | + | + | + |
| 13 | MNE | + | + | + | + | + |
| 14 | SBE | - | +/- | + | + | - |
| 15 | RHA | + | + | + | + | + |
| 16 | DUL | - | + | - | - | + |
| 17 | INO | + | + | + | + | + |
| 18 | MAN | + | + | + | + | + |
| 19 | SOR | + | + | + | + | + |
| 20 | MDM | - | + | + | + | + |
| 21 | MDG | + | + | + | + | + |
| 22 | NAG | + | + | + | + | + |
| 23 | AMY | + | + | + | + | + |
| 24 | ARB | + | + | + | + | + |
| 25 | ESC | + | + | + | + | + |
| 26 | SAL | + | + | + | + | + |
| 27 | CEL | + | + | + | + | + |
| 28 | MAL | + | + | + | + | + |
| 29 | LAC | - | + | + | + | + |
| 30 | MEL | - | + | + | + | + |
| 31 | SAC | + | + | + | + | + |
| 32 | TRE | + | + | + | + | + |
| 33 | INU | + | + | + | + | + |
| 34 | MLZ | - | + | - | + | - |
| 35 | RAF | + | + | + | + | + |
| 36 | AMD | + | + | + | + | + |
| 37 | GLYG | + | + | + | + | + |
| 38 | XLT | + | +/- | - | - | + |
| 39 | GEN | + | + | + | + | + |
| 40 | TUR | - | + | + | + | + |
| 41 | LYX | + | + | - | + | + |
| 42 | TAG | + | + | + | + | + |
| 43 | DFUC | + | - | - | - | - |
| 44 | LFUC | + | + | - | + | + |
| 45 | DARL | + | + | - | + | + |
| 46 | LARL | + | + | - | + | + |
| 47 | GNT | - | + | - | - | - |
| 48 | 2KG | + | +/- | + | - | - |
| 49 | 5KG | - | + | - | + | + |
| API 20 E |  |  |  |  |  |  |
| 1 | ONPG | - | - | + | + | + |
| 2 | ADH | + | - | + | + | + |
| 3 | LDC | + | - | + | + | - |
| 4 | ODC | + | + | + | + | + |
| 5 | CIT | - | - | + | + | + |
| 6 | H2S | - | - | - | - | - |
| 7 | URE | - | - | + | - | - |
| 8 | TDA | - | - | - | - | - |
| 9 | IND | - | - | - | - | - |
| 10 | VP | + | + | + | + | + |
| 11 | GEL | + | + | - | + | + |
| 12 | GLU | + | + | + | + | + |
| 13 | MAN | + | + | + | + | + |
| 14 | INO | + | + | + | + | + |
| 15 | SOR | + | + | + | + | + |
| 16 | RHA | - | - | - | - | - |
| 17 | SAC | + | + | + | + | + |
| 18 | MEL | - | - | - | - | - |
| 19 | AMY | + | + | + | + | + |
| 20 | ARA | + | + | + | + | + |

**Table S5.** Enzymatic activities of the selected aerobic spore-forming bacterial strain determined using the API ZYM gallery

| API ZYM | Enzymatic test | F12 | F2 | F8 | F9 | F11 |
| --- | --- | --- | --- | --- | --- | --- |
| Control | Temoin | - | - | - | - | - |
| 2 | Phosphatase alcaline | + | + | + | - | - |
| 3 | Estérase (C 4) | + | + | + | - | + |
| 4 | Estérase Lipase (C 8) | + | + | + | - | - |
| 5 | Lipase (C 14) | - | - | - | - | - |
| 6 | Leucine arylamidase | + | + | + | + | + |
| 7 | Valine arylamidase | - | - | - | - | - |
| 8 | Cystine arylamidase | - | - | - | - | - |
| 9 | Trypsin | - | - | - | - | - |
| 10 | Chymotrypsine | - | - | - | - | - |
| 11 | Phosphatase acide | + | + | + | - | - |
| 12 | Naphtol-AS-BI- phosphohydrolase | + | + | + | + | - |
| 13 | α-galactosidase | - | - | - | - | - |
| 14 | β-galactosidase | - | - | - | - | - |
| 15 | β-glucuronidase | - | - | - | - | - |
| 16 | α-glucosidase | - | - | + | - | - |
| 17 | β-glucosidase | + | - | + | - | - |
| 18 | N-acetyl-beta- glucoseamidase | - | - | - | - | - |
| 19 | α-mannosidase | - | - | - | - | - |
| 20 | α-fucosidase | - | - | - | - | - |
| 21 | α-amylase | + | + | + | + | + |
| 22 | Keratinase | + | - | + | + | + |
| 23 | Cellulase | - | + | + | + | - |

**Table S6.** Digital DNA–DNA hybridization (dDDH) values between the genome of *Bacillus* sp. F11 and those of related type strains, calculated using the Genome-to-Genome Distance Calculator (GGDC), available on the DSMZ server.


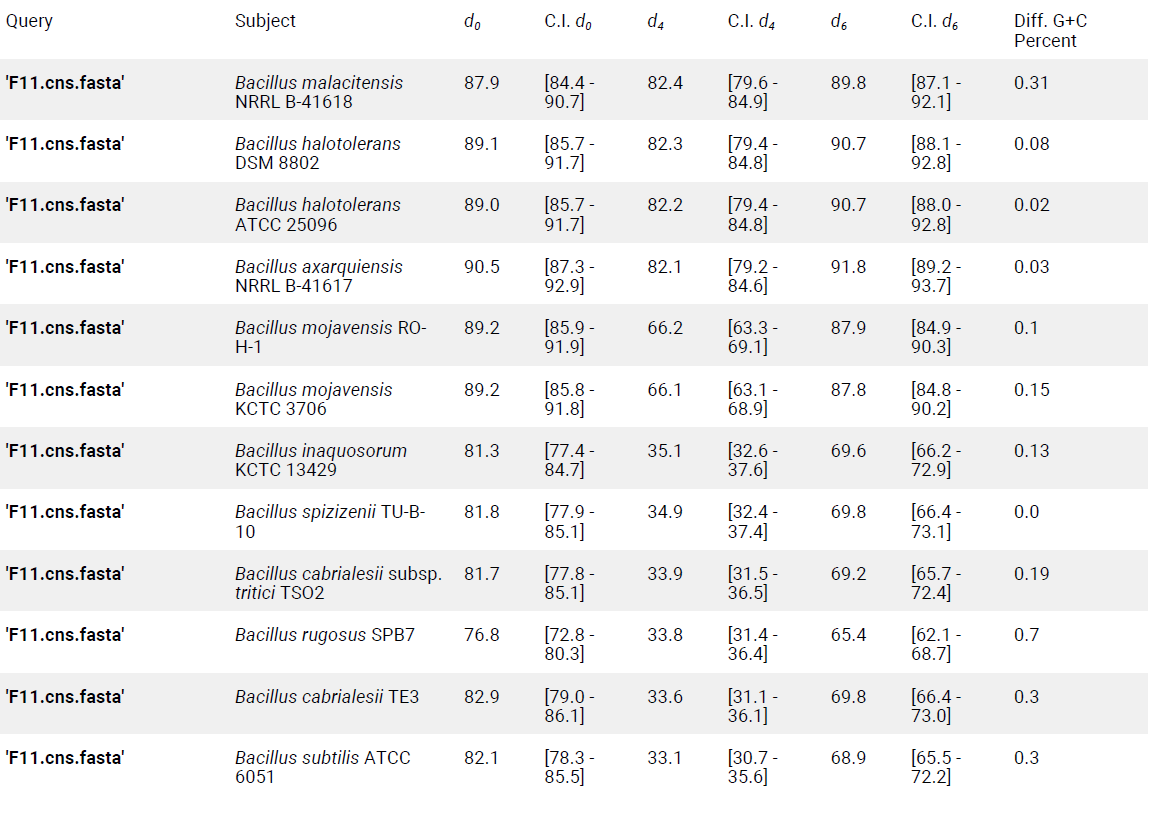


**Table S7.** Average Nucleotide Identity (ANI) values between *B.halotolerans F11* and closely related *Bacillus* species, calculated using FastANI.

|  | *B.halotolerans F11* | B.malacitensis NRRLB41618 | *B.axarquinsis* NRRLB41617 | *B.halotolerans* ATCC25096 | *B. mojavensis* ROH1 |
| --- | --- | --- | --- | --- | --- |
| *B.halotolerans F11* | - | 97.7887 | 97.7598 | 97.7591 | 95.7207 |
| *B.malacitensis* NRRLB41618 | - | - | 99.1014 | 99.012 | 95.5406 |
| *B.axarquinsis* NRRLB41617 | - | - | - | 99.0745 | 95.4911 |
| *B.halotolerans* ATCC25096 | - | - | - | - | 95.5231 |

**Table S8.** Geographic origin of the 24 *Bacillus halotolerans* strains used in this study.

| **Clade I** | | |
| --- | --- | --- |
| **Strains** | **Geographic Location** | **The nature of samples** |
| **F11** | Algeria, Laghouat | Soil collected from Oued Mzil |
| **OM-41** | Moroccan | Olive rhizospheres |
| **KKD1** | China:Qinghai | Saline-alkali rhizosphere soil of Androsace umbellata in Hoh Xil |
| **MBH1** | USA: Arkansas, Arkadelphia | Mixed forest with bamboo |
| **P1** | USA: Wyoming | Pisolithus arhizus, Geothermal soil |
| **Tehuacan_S4** | Mexico: Tehuacan, Puebla | Smidesertic area with dry sandy soils of a peach tree (5 cm deep) |
| **BCP32** | deep-sea sediments | Dohrn Canyon in the Gulf of Naples (Italy), |
| **NRRL B-41618** | Spain | Velez revier |
| **HMB20199** | China: Baoding | Plant |
| **ATCC 25096** | Morocco | Arid soil |
| **XYK2-4** | China: Weinan town, Shaanxi Province | Rhizosphere soil |
| **XE48** | Indian Ocean: West Indian Ocean | Low temperature surface sediment |
| **B13** | China:Shandong | Sweet potato rhizosphere soil |
| **S-5** | China:Shandong | Soil |
| **MEC_B301** | China:Beijing | Soil |
| **NRRL B-41617** | Spain | River Velez |
| **Clade II** | | |
| **DY299** | China: Inner Mongolia | Soil |
| **KF17** | China: Kaifeng, Henan Province | Rhizosphere soil |
| **SW207** | China: Jilin Province, Changchun | Sus scrofa from gut content sample |
| **MEC_B334** | China:Shandong | American ginseng Root |
| **Q2H2** | China: Inner Mongolia | Endophyte from hree year continuous cropping of potato root |
| **LN2** | China: Longnan, Gansu Province | Rhizosphere soil |
| **XH-1** | China | Unkown |
| **ZB201702** |  | Dry salty rhizosphere soil of maize |

**Table S9.** Functional characterization of strain-specific genes identified in *B. halotolerans* F11 through pangenome analysis.

| **Genes** | **Predicted Function** | **Adaptive Role for Strain F11** |
| --- | --- | --- |
| **YvyC/ fliD** | Involved in flagellar assembly and function, contributing to bacterial motility and chemotactic responses. | Reflect an adaptive advantage of *B. halotolerans* F11, enabling enhanced movement toward nutrient sources |
| **ydeA** | Encodes a membrane transporter involved in the efflux of small molecules. | Contributes to multidrug and sugar efflux activity, potentially helping regulate intracellular sugar levels and confer resistance to molecular toxicity. |
| **fenA and /fenC** | Involved in biosynthetic potential to produce fengycin-like lipopeptides. | Contribute to antimicrobial activity |
| **yxkC** | Involved in flagellar assembly and function, contributing to bacterial motility and chemotactic behavior. | Reflect an adaptive advantage of *B. halotolerans* F11, enabling enhanced movement toward nutrient sources |
| **yjdB** | Induced under phosphate starvation conditions in a PhoR-dependent manner. | Contribute to environmental adaptation under phosphate-limited conditions, |
| **nagP** | part of the PTS system and encodes a transporter responsible for the uptake of N-acetylglucosamine (GlcNAc) and/or glucosamine, | Facilitates the import and phosphorylation of GlcNAc and glucosamine |
| **rhaS** | Encodes a helix-turn-helix (HTH) transcriptional activator involved in the regulation of rhamnose metabolism. | Facilitates adaptation to variable sugar availability in the soil by enabling the utilization of L-rhamnose derived from plant or microbial sources |
| **eamA_1** | Aamino-acid metabolite efflux pump | Removes excess or potentially toxic metabolites (such as cysteine, O-acetyl-L-serine, or other amino acid derivatives), thereby supporting cellular detoxification and adaptation to changing environmental conditions. |
| **group_484** | Unkown | Unkown |
| **group_529** | Unkown | Unkown |
| **group_718** | Unkown | Unkown |
| **group_1014** | Unkown | Unkown |
| **group_3309** | Unkown | Unkown |
| **group_905** | Unkown | Unkown |
| **group_107** | Unkown | Unkown |

**Table S10.** Overview of BiG-SCAPE analysis results of *B. halotolerans* F11 biosynthetic gene clusters (BGCs)

|  | **BGCs** | **Gene cluster families** | **Singleton** | **Link** |
| --- | --- | --- | --- | --- |
| **NRPS** | 58 | 20 | 11 | 105 |
| **RiPPS** | 41 | 26 | 20 | 38 |
| **PKSother** | 45 | 20 | 15 | 90 |
| **PKS-NRP Hybrids** | 16 | 7 | 4 | 44 |
| **Terpene** | 32 | 8 | 3 | 89 |
| **Others** | 61 | 19 | 7 | 96 |
| **Total** | 253 | 100 | 60 | 462 |

**Table S11.** Structural Characterization of Fengycin R10 BGC

| **Locus tag** | **Assigned gene** | **Modules** | **Predicted amino acid** | **Identity (%)** | **NCBI Reference Sequence** |
| --- | --- | --- | --- | --- | --- |
| ctg1_4110 | FenA | M1 | L-Glu | 96.20% | WP_202647342.1 |
|  |  | M2 | D-Orn |  |  |
| ctg1_4111 | FenB | M3 | D-Tyr | 98.50% | WP_400153640.1 |
|  |  | M4 | D-Thr |  |  |
| ctg1_4112 | FenC | M5 | L-Glu | 97.90% | WP_369888579.1 |
|  |  | M6 | D-X |  |  |
| ctg1_4113 | FenD | M7 | L-Pro | 98.25% | WP_322008948.1 |
|  |  | M8 | L-X |  |  |
|  |  | M9 | D-Tyr |  |  |
| ctg1_4114 | FenE | M10 | L-Ile | 94.65% | WP_326202552.1 |

**Table S12.** Antimicrobial activity (inhibition zones, mm) of *B. halotolerans* F11 against various pathogenic microorganisms, evaluated in five different culture media using the agar plug method

|  | SM | YIM 38 | ISP2 | NA | TSA |
| --- | --- | --- | --- | --- | --- |
| *P. aeruginosa* | 10 | 12 | 13 | 29 | 11.5 |
| *E. coli* | 8 | - | - | - | - |
| *Y. enterocolitica* | - | 9.5 | - | - | - |
| *B. cereus* | - | - | - | - | 9.5 |
| *S. aureus* | 20.5 | - | 17 | - | - |
| *M. luteus* | 30 | 30 | 30 | - | 30 |
| *K. pneumoniae* | - | - | - | - | - |
| *C. albicans 10* | 23 | - | 19.5 | - | - |
| *C. albicans 26* | 24 | . | 19 | - | - |

‘-’**:** No activity detected; **SM**: Soybean Medium; **YIM38**: Methylophila Medium; **ISP2**: The International Streptomyces Project (ISP) Medium 2; **NA**: Nutrient Agar. **TSA**: Tryptic soy agar

**Table S13.** Minimum inhibitory concentration (MIC, µg/mL) of the crude extract obtained from *B. halotolerans* F11 cultured in ISP2 medium against different pathogenic microorganisms, determined by serial dilution in 96-well microplates

| *Tested Strain* | *S.*  *aureus* | *Y.*  *enterocolitica* | *S.*  *typhi* | *K.*  *pneumoniae* | *E.*  *coli* | *P.*  *aeruginosa* | *C.*  *albicans10* | *C.*  *albicans26* |
| --- | --- | --- | --- | --- | --- | --- | --- | --- |
| F11 | 1.66 | 1.66 | 1.66 | - | - | - | 1.66 | 1.66 |

‘-’: No activity

**Table S14**. Number of metabolites synthesized by *Bacillus halotolerans* F11 under the applied culture conditions, showing the total and identified metabolites (BGI untargeted metabolomics report).

| **Number of Total metabolites** | **Number of identified metabolites** |
| --- | --- |
| **9719** | **3453** |

| 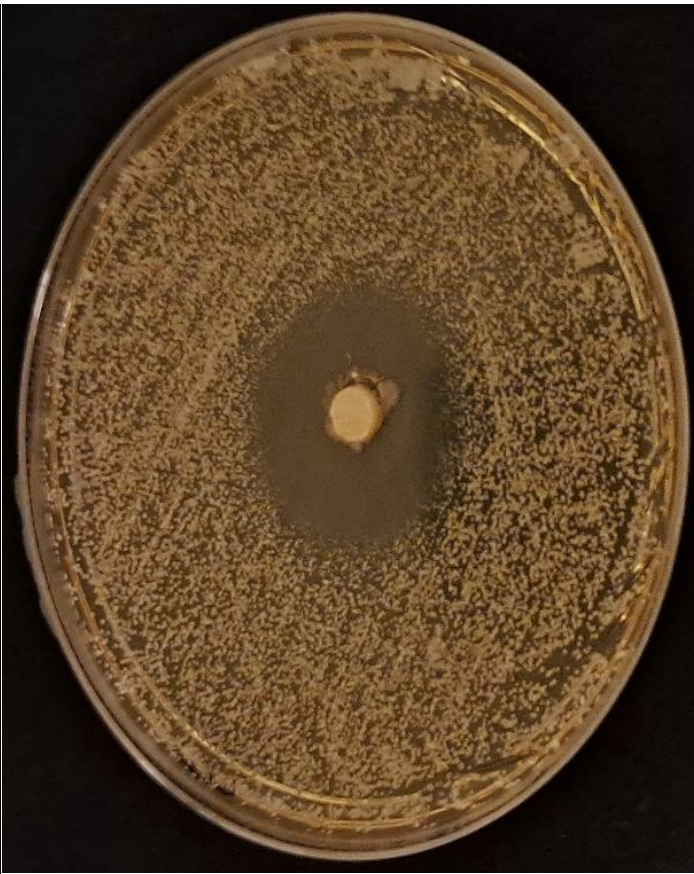 | 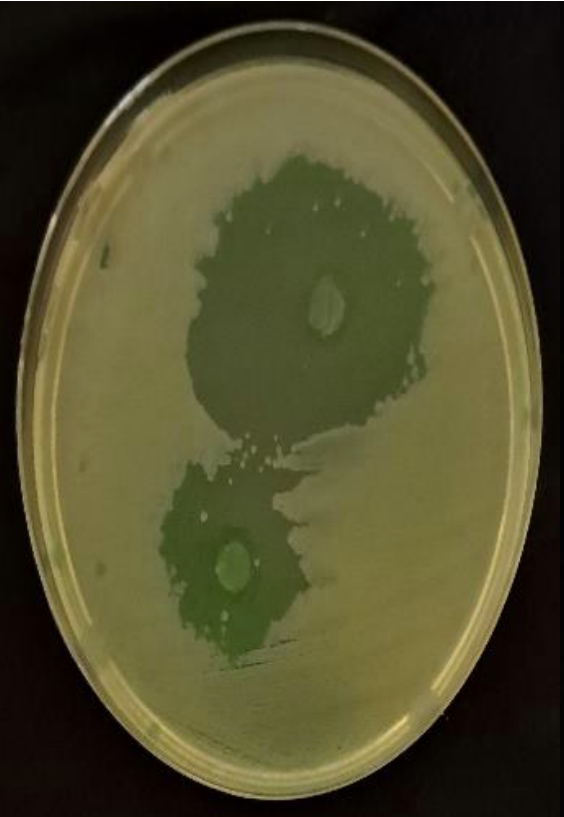 | 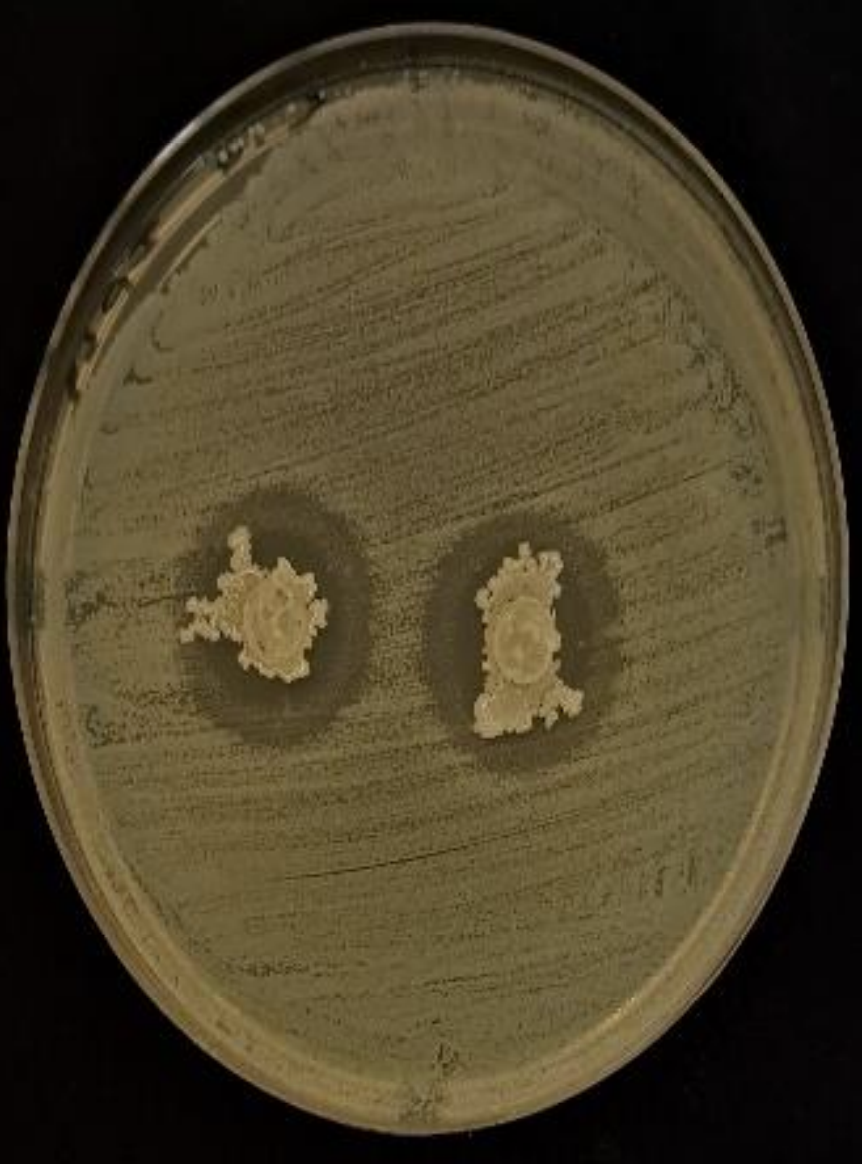 |
| --- | --- | --- |
| ***Candida albicans*** | ***Pseudomonas aeruginosa*** | ***Staphylococcus aureus*** |

**Figure S1.** Antibacterial activity of some active *Bacillus* sp. isolates against tested bacteria.


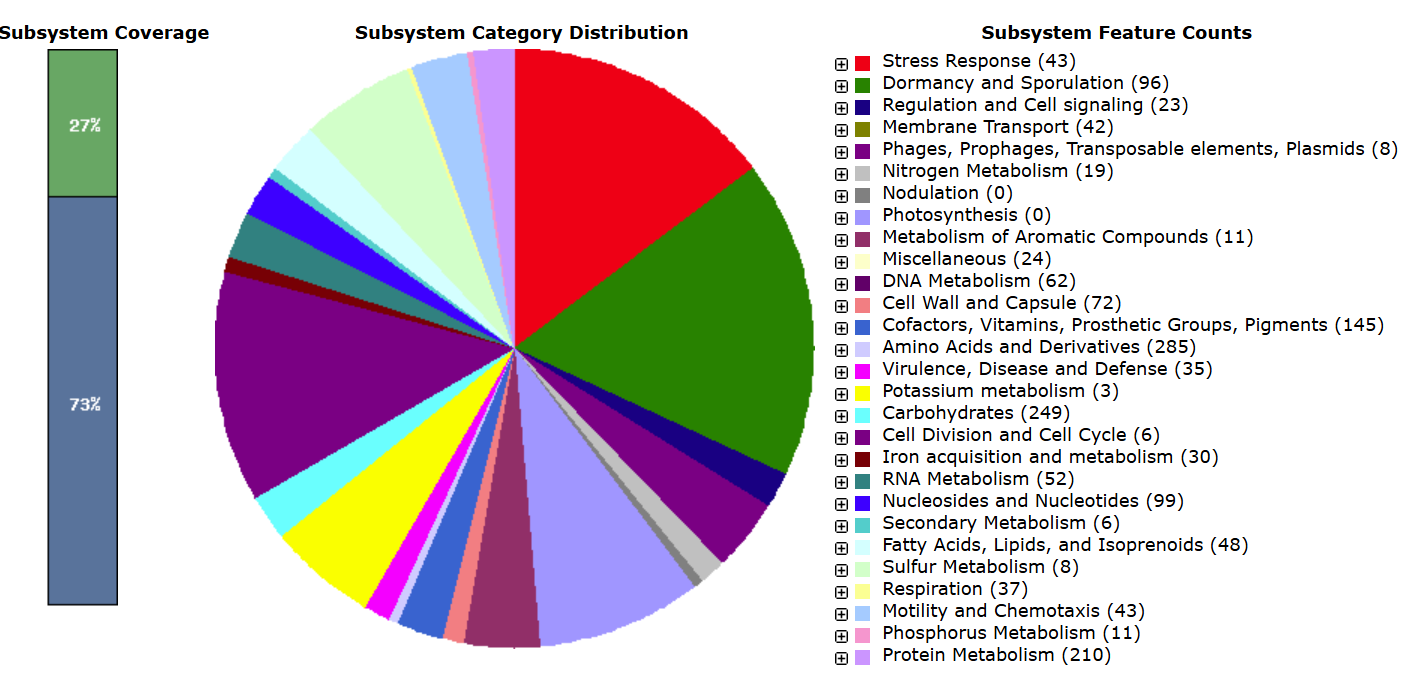


**Figure S2.** Distribution of Annotated Genes Across Subsystem Categories in the *Bacillus halotolerans* F11 Genome

**
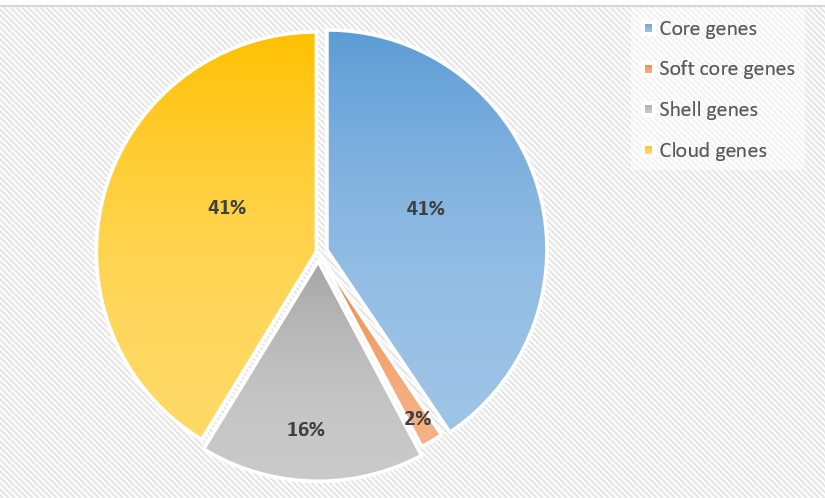
**

**Figure S3.** Cumulative pangenome statistics of 24 *Bacillus halotolerans genomes* from different strains, showing the distribution of core, accessory, and unique genes.


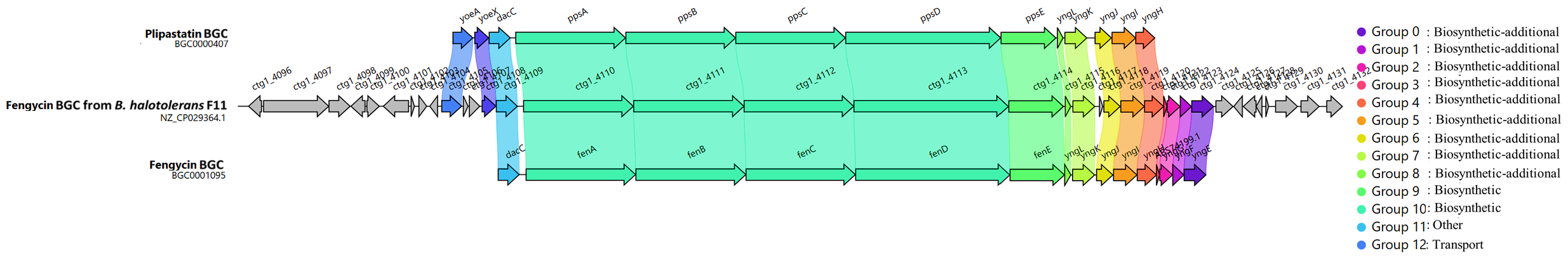


**Figure S4.** Comparative Synteny Analysis of the Fengycin BGC from *B. halotolerans* F11 and two Well-Characterized Reference fengycin and plipastatin Clusters


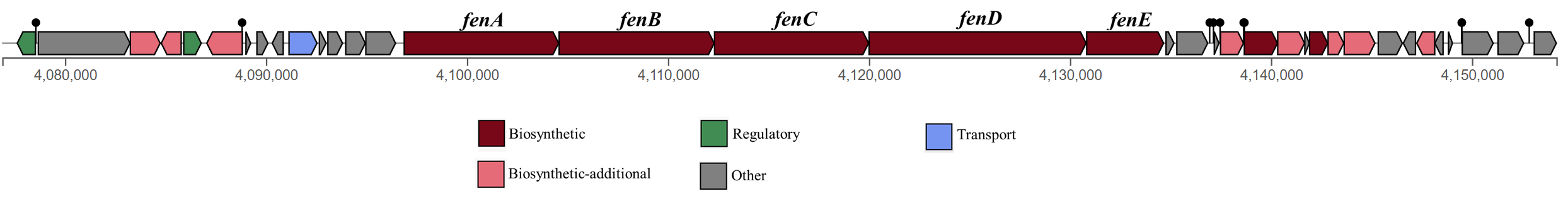


**Figure S5.** Complete Fengycin-Like Biosynthetic Gene Cluster in *B. halotolerans* F11 Reveals Canonical Gene Organization

**
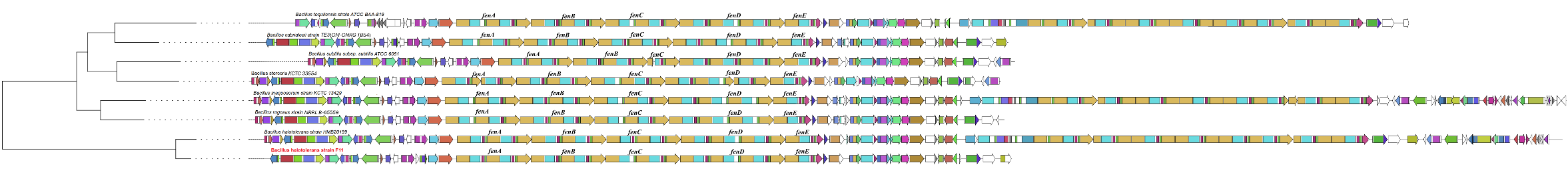
**

**Figure S6.** Protein-level synteny analysis of fengycin-type BGCs across *Bacillus* species using CORASON
